# Aggregation Induced Effects on the Nonradiative Recombination Dynamics of Inverted Singlet–Triplet Heptazine-Based Materials

**DOI:** 10.1021/acs.jpca.5c00709

**Published:** 2025-06-04

**Authors:** Laure de Thieulloy, Robson S. Oliboni, Piotr de Silva, Luis G. C. Rego

**Affiliations:** † Department of Energy Conversion and Storage, 5205 Technical University of Denmark, 2800 Kongens Lyngby, Denmark; ‡ Department of Chemistry, 37902 Universidade Federal de Pelotas, Pelotas, Rio Grande do Sul 96010-900, Brazil; § Department of Physics, Universidade Federal de Santa Catarina, Florianópolis, Santa Catarina 88040-900, Brazil

## Abstract

Heptazine (C_6_N_7_H_3_) serves as the core unit for various molecular and self-assembled compounds, finding applications in photocatalysis and optoelectronics. Some of its derivatives, such as melem and melon, are known to exhibit thermally activated delayed fluorescence (TADF). However, hindered by their insolubility and chemical inertness, a comprehensive understanding of the molecular mechanisms governing the photorelaxation dynamics of these compounds remains a matter of investigation. In this work, we present the first excited-state nonadiabatic simulations of heptazine-based molecules and aggregates, aiming to elucidate the role of nonradiative pathways in their photorelaxation processes. Our results reveal that isolated heptazine and melem (C_6_N_10_H_6_) molecules return to the ground state via conical intersections within subpicosecond time scales following photoexcitation. In contrast, melem aggregation, driven by strong hydrogen bonding, markedly suppresses nonradiative photorelaxation through two mechanisms: (i) intermolecular charge transfer, which reduces the likelihood of electron–hole recombination via internal conversion, and (ii) molecular packing, which prevents ring deformation, thus reducing the occurrence of conical intersections. These findings suggest that, in addition to being a TADF material, melem’s high photoluminescence quantum yield is further enhanced by aggregation-induced emission (AIE). Additionally, the findings provide valuable insights into the mechanisms underlying nonradiative recombination in organic solar cells.

## Graphical abstract

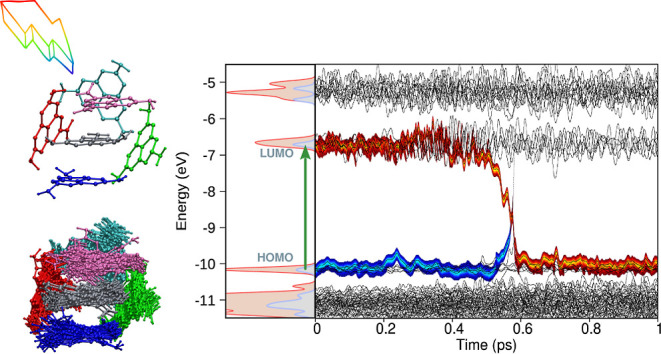

## 1 Introduction

Heptazine (also known as *s*-heptazine or tri*s*-triazine) is a carbon-nitride molecular compound with the formula C_6_N_7_H_3_, shown in Figure 1a, which serves as the core unit for various molecular and polymeric materials, self-assembled structures, crystals, and 2D-like organic semiconductors.^1–3^ Heptazine (HTZ), along with related nitrogen-based compounds such as melem and melon, have been known for their exceptional thermal stability, low solubility, and limited chemical reactivity. More recently, interest in the electronic properties of heptazine-based materials has surged. In 2008, g-C_3_N_4_, a heptazine-based polymeric material, was identified as a metal-free photocatalyst capable of producing hydrogen from water under visible light.^4,5^ Heptazine-based molecules have also attracted attention in optoelectronics, following reports that an OLED employing the heptazine-based compound HAP-3MF as the emitter exhibited high photoluminescence due to thermally activated delayed fluorescence (TADF).^6,7^ Furthermore, theoretical studies suggest that heptazine and related compounds exhibit an inversion of the lowest singlet and triplet excited states in violations of Hund’s rule.^8–11^ Together with their symmetry-forbidden S_1_ → S_0_ radiative transition, it raises questions about the mechanisms underlying their observed luminescence.^12^ Despite these advances, systematic investigations into the excited-state dynamics of heptazine-based molecules are still absent. Existing computational studies have primarily focused on mapping potential energy profiles of excited states to better understand their photophysical properties and reaction pathways. On the other hand, molecular design of inverted singlet–triplet emitters focuses predominantly on increasing their oscillator strengths,^13–16^ while the concomitant effect on nonradiative relaxation is typically not addressed.

**Figure 1:**
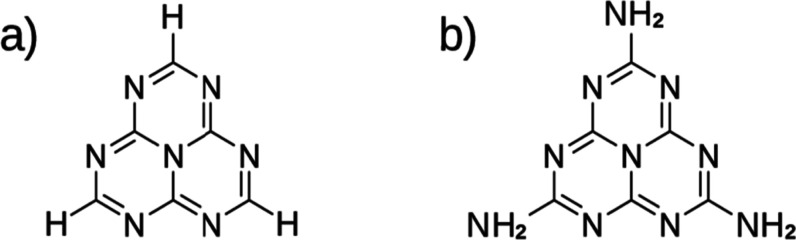
Schematic representations of the heptazine (HTZ) (a) and melem (MLM) (b) molecular structures.

Experimentally, insights into the photoluminescence (PL) of carbon-nitride compounds have been advanced through studies of melem (C_6_N_7_(NH_2_)_3_), Figure 1b, the simplest heptazine derivative.^17,18^ These investigations revealed that the PL quantum yield (PLQY) in oxygen-saturated solutions is approximately 10% of that in oxygen-free solutions, clearly indicating that melem exhibits TADF. Most of its PLQY originates from upconversion from the triplet to the singlet state, with an activation energy of approximately 31 meV.^17^ Furthermore, crystalline melem was found to have a PLQY roughly seven times higher than that of melem oligomers (e.g., tetramers) and amorphous melon, underscoring the critical role of crystallinity and molecular packing in the PL process. This behavior exemplifies aggregation-induced emission (AIE), where emission is enhanced in the aggregated or solid state while being weak or negligible in dilute solutions.^19,20^

The rigidity of the heptazine core has been argued to suppress nonradiative decay pathways, thereby enabling photoluminescence. In this study, we propose instead that molecular aggregation plays a more critical role in inhibiting nonradiative decay. Experimental evidence shows that small aromatic amino compounds often exhibit short lifetimes, typically on the subnanosecond to subpicosecond time scale.^21–27^ Amino substitution induces twisting and wagging deformations in the chromophore structure, facilitating the formation of conical intersections with the ground state.^26,28–33^ These effects are substantial, with quenching rate constants varying over several orders of magnitude.^34^

Herein, we investigate the photorelaxation dynamics of heptazine molecules. We perform large-scale excited-state nonadiabatic molecular dynamics simulations on isolated heptazine (HTZ, C_6_N_7_H_3_), melem (MLM, C_6_N_7_(NH_2_)_3_), and a six-molecule melem aggregate (6-MLM) in the gas phase to compare their excited-state responses. Our simulations reveal that isolated molecules undergo nonradiative decay on picosecond and subpicosecond time scales. The substitution of terminal amino groups in HTZ to form MLM significantly accelerates nonradiative decay relative to HTZ. However, MLM molecules readily form stable aggregates via strong N···NH hydrogen bonds. In these aggregates, photorelaxation is markedly suppressed due to two key factors: (i) inter-MLM charge transfer, which reduces the likelihood of electron–hole recombination through nonradiative pathways, and (ii) molecular packing, which constrains ring deformations responsible for nonradiative decay.

The present results reveal that, in addition to being a TADF material, the high photoluminescence quantum yield of melem is enhanced by aggregation-induced emission (AIE). More broadly, these findings on the impact of molecular aggregation on nonradiative recombination rates can provide insights into the mechanisms underlying voltage losses in state-of-the-art organic solar cells.^35–37^

## 2 Methods

### 2.1 Theoretical Method

To perform the simulations, we implemented an efficient semiempirical version of the Ehrenfest method with Coherent Switches with Decay of Mixing (CSDM).^38–42^ The details of this theoretical method are provided in the Supporting Information. The CSDM procedure combines the principles of the Ehrenfest method with the Fewest Switches Surface Hopping (FSSH) algorithm into a single approach. Essentially, CSDM adds a phenomenological decoherence term to the time-dependent Schrödinger equation for the electrons, which induces the decoherence of the electronic wave function to a pointer state associated with a specific potential energy surface (PES). The kinetics of the pointer state is controlled by Tully’s fewest switches algorithm.^43^ This approach naturally addresses one of the main deficiencies of the mean-field Ehrenfest method, which is the propagation of nuclear trajectories on an average (mean-field) potential energy surface.

The quantum-classical mixed approach used herein is presented next.^44,45^ The time-dependent Schrödinger equation (TDSE) is solved for the electronic degrees of freedom

1 $$i\hbar \frac{\partial }{\partial t}|\Psi \left(r;t\right)\rangle ={Ĥ}_{el}\left(R_{t}\right)|\Psi \left(r;t\right)\rangle$$

where **r** designates the electronic coordinates, **R**_*t*_ ≡ **R**(*t*) are the time-dependent nuclear coordinates and ${Ĥ}_{el}\left(R_{t}\right)$ is the time-dependent extended Hückel Hamiltonian for an instantaneous molecular configuration. We use the Generalized Amber Force Field (GAFF2, version 2.2.20) to describe the nuclear dynamics

2 VGSMM({R})=∑bondsKb(R−R0)2+∑anglesKθ(θ−θ0)2+∑dihedralsVn(1+cos⁡(nϕ−γ)n+∑i<j4εij[(σijRij)12−(σijRij)6]+∑i<jqjqi4πϵ0Rij

where **R** is the atomic position and **R**_*ij*_ = |**R**_*i*_ – **R**_*j*_| is the distance between atoms *i* and *j*, which have fixed partial charges *q*_*i*_ and *q*_*j*_. Likewise, θ and ϕ are the angular and dihedral torsional variables. The parameters **R**_0_ and θ_0_ are the equilibrium bond length and angle; *K*_b_, *K*_θ_ and *V*_*n*_ (where *V*_*n*_ = v_*n*_/2, as it appears in some documentation^46^) are the intramolecular FF parameters, and ε_*ij*_ and σ_*ij*_ are the Lennard-Jones parameters. The FF parameters are described as Supporting Information.

To perform the nonadiabatic nuclear dynamics, we extend the ground-state molecular mechanics (MM) framework to the excited-state, where the nuclear dynamics are governed by the classical equations of motion

3 Ṙ=P/M

4 $$Ṗ=-\nabla_{R}{\left\langle \Psi \left(r;t\right)\left|V\left(r,R\right)\right|\Psi \left(r;t\right)\right\rangle }_{r}$$

where we approximate the potential energy term as follows

5 $${\left\langle \Psi \left(r;R,t\right)\left|V\left(r,R\right)\right|\Psi \left(r;R,t\right)\right\rangle }_{r}\approx V_{GS}^{MM}\left(R\right)+V_{EH}\left[\Psi^{el}\left(R,t\right),\Psi^{hl}\left(R,t\right)\right]$$

In this expression, the excited-state interatomic potential is decomposed into the ground-state force field potential, *V*_GS_^MM^, and an excitation-induced correction term, *V*_EH_[Ψ^el^,Ψ^hl^]. The rationale behind this approximation is that the classical force field *V*_GS_^MM^ accurately captures the forces arising from the occupied orbitals. The term *V*_EH_, a functional of the photoexcited electron and hole wave functions, is introduced to account for the excited-state charge redistribution. It gives rise to nonadiabatic Hellmann–Feynman–Pulay forces, which serve as excitation-induced corrections to the ground-state potential, effectively capturing the back-reaction of the evolving electronic structure on the nuclear dynamics. This approach allows us to retain fixed ground-state charges while still incorporating essential excited-state effects, and it ensures conservation of the total energy of the system (classical + quantum) throughout the dynamics. The term *V*_EH_ is calculated on-the-fly as

6 $$V_{EH}\left[\Psi^{el}\left(R,t\right),\Psi^{hl}\left(R,t\right)\right]=Tr\left[\rho^{EH}\left(R,t\right)H\left(R_{t}\right)\right]$$

where ρ^EH^ = |Ψ^el^⟩⟨Ψ^el^| – |Ψ^hl^⟩⟨Ψ^hl^| is the electron–hole density matrix. In the excited-state, the force produced on atom *N* by an e–h excitation is given by

7 $$F_{N}=-\nabla_{N}V_{EH}\left[\Psi^{el}\left(R,t\right),\Psi^{hl}\left(R,t\right)\right]$$

where ∇_N_ ≡ ∇_**R**_N__. This force is responsible for the electronic back-reaction on the classical degrees of freedom and it gives rise to nonadiabatic nuclear dynamics effects.^44,45,47^ In this approach, the total energy of the excited system consists of the overall classical energy of the nuclei (kinetic plus potential) as given by the molecular mechanics (MM) formalism, along with the quantum energy of the excited electron–hole pair, given by *E*_QM_ = Tr­[ρ^EH^ **H**].

The implementation details of the CSDM method within the semiempirical framework are provided in the Supporting Information. All semiempirical calculations were conducted using the DynEMol simulation package.^48^

### 2.2 System Preparation

We used the Amber force field^46^ with GAFF2 parameters (version 2.2.20, March 2021)^49^ to describe the ground-state molecular structure of the heptazine compounds. Atomic charges were assigned using the AM1-BCC model^50^ within the Antechamber package.^51^ After testing various force field parameters and atomic charge models, the combination of GAFF2 parameters with AM1-BCC charges showed the best agreement with ground-state geometries obtained from density functional theory (DFT) calculations.

To validate the force field, we compared the ground-state optimized geometry of the heptazine molecule with DFT results using the long-range corrected hybrid functional wB97xD^52^ and the 6-31g­(d,p) basis set, with the ORCA program.^53^ Figure S1 shows an excellent match between the geometries optimized by the classical force field and those obtained from DFT for the heptazine molecule, as well as for the monomer, dimer, and trimer of the melem compound; additional details are provided in the Supporting Information.

Before performing the excited-state simulations, we parametrized the extended Hückel model Hamiltonian for the heptazine (C_6_N_7_H_3_) using Slater-type orbitals (STOs). The e-Hückel model Hamiltonian is defined as $H_{ij}=\frac{K_{ij}}{2}\left(V_{i}+V_{j}\right)S_{ij}$ , where *V*_*i*_ is associated with the valence-state ionization potentials of a given atomic species, *K*_*ij*_ is an adjustable coupling parameter between atomic orbitals, and *S*_*ij*_ is the associated overlap matrix element between STOs. For the C_6_N_7_H_3_ molecule, we used STOs: 2s and 2p for Carbon and Nitrogen, and 1s for Hydrogen. For the parametrization procedure, we employed our Adaptive Genetic Algorithm with Symmetry Descriptors, which was calibrated to reproduce the charge distribution of the frontier molecular orbitals (MOs) and the associated excitation energy calculations based on algebraic diagrammatic construction to the second order, ADC(2) and the cc-pVDZ basis set, performed with TURBOMOLE,^54^ as detailed in the Supporting Information. The choice of this relatively expensive electronic structure method was dictated by the qualitative failure of time-dependent DFT, which would otherwise be a natural choice, in capturing the partially double excited character of the excited states in molecules exhibiting singlet–triplet inversion.^8,9^ The isosurfaces of the frontier molecular orbital wave functions are shown in Figure 2, comparing the Kohn–Sham MOs obtained using the DFT-wB97xD/6-31g­(d,p) method (green-brown) with those from the parametrized extended-Hückel model Hamiltonian (blue-red). The comparison of the excitation energy calculations is shown in Table 1. The same set of parameters were also applied to describe the melem compounds, yielding similarly accurate results, as detailed in the Supporting Information. The electronic spin was not taken into account, so that the dynamics is assumed to be confined to the singlet space.

**Figure 2:**
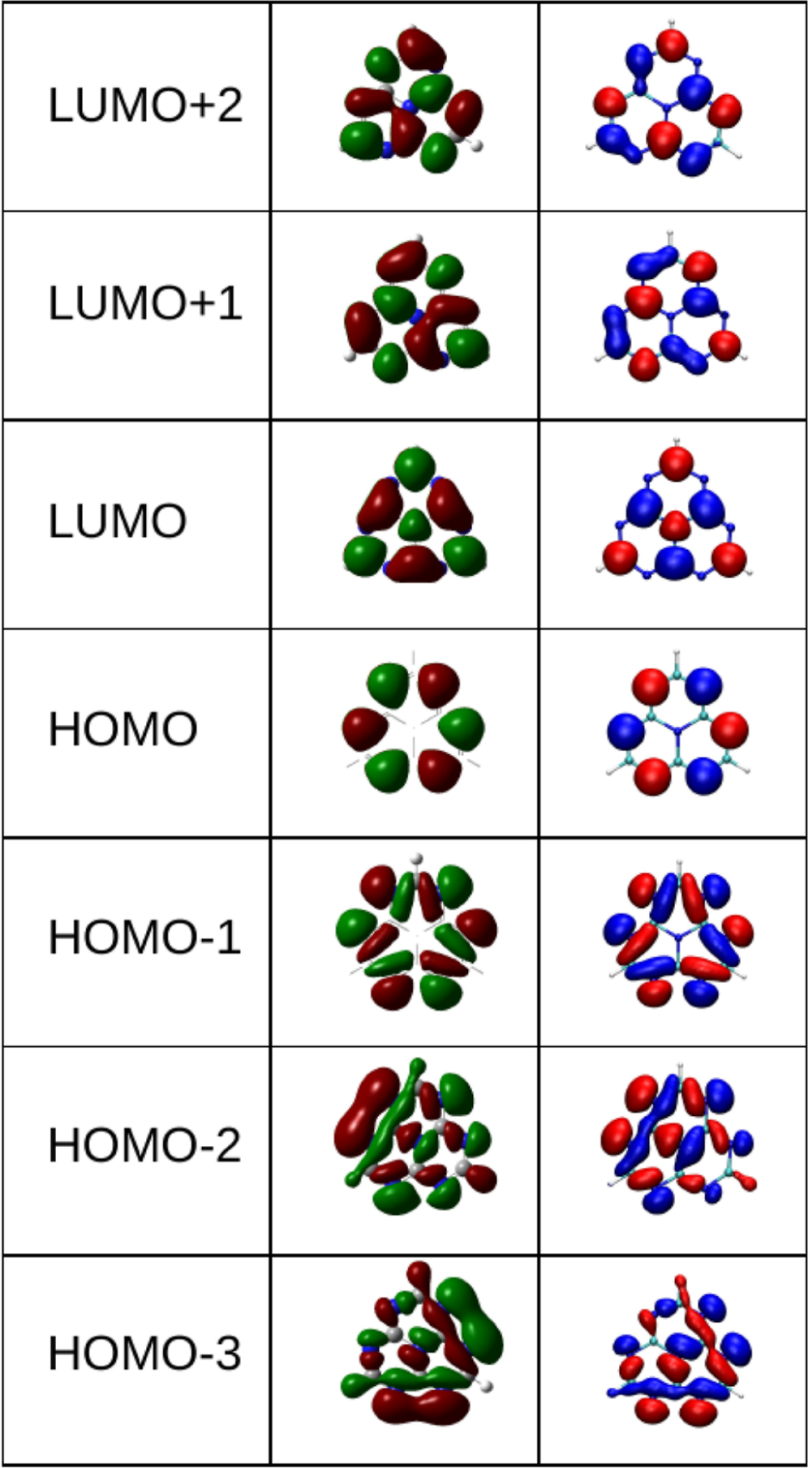
Isosurfaces of the frontier molecular orbital wave functions for HTZ: Kohn–Sham MOs obtained using the DFT-wB97xD/6-31g­(d,p) method (green-brown) and the parametrized e-Hückel model Hamiltonian (blue-red).

**Table 1:** Electronic Transitions with Corresponding Excitation Energies and Oscillator Strengths (*f*) for the C_6_N_7_H_3_ Molecule in the Optimized Ground-State Geometry

TURBOMOLE/ADC(2) cc-pVDZ			e-Hückel model hamiltonian	
transition	*E* (eV)	*f*	MO transition	*E* (eV)
S_0_ → S_1_	2.70	0	H → L	2.769
S_0_ → S_2_	3.78	0	H – 3 → L	3.698
S_0_ → S_3_	3.87	0	H – 2 → L	3.777
S_0_ → S_4_	3.87	0	H – 1 → L	3.778
S_0_ → S_5_	4.65	0.273	H → L + 1	4.656
S_0_ → S_6_	4.65	0.273	H → L + 2	4.661

Our ADC(2) calculations reveal that the first singlet and triplet excited states of the HTZ molecule are both dominated by a HOMO-to-LUMO transition, corresponding to a π → π* electronic excitation. The HOMO is predominantly localized on the peripheral nitrogens, whereas the LUMO is localized on the peripheral carbons and the central nitrogen of the molecule. The S_0_ → S_1_ transition is predominantly a HOMO–LUMO excitation, which is symmetry-forbidden within the Franck–Condon approximation. Similarly, the bright S_5_ and S_6_ excitations listed in Table 1, can be accurately described as π* – π single particle-excitations from the HOMO to the degenerate LUMO + 1 and LUMO + 2 molecular orbitals.^8,10^ This supports our use of a single-particle model Hamiltonian to describe the excited states of heptazine. However, the contribution of double excitations is essential for accurately capturing the energetics of heptazine derivatives. In our model, their effect is effectively incorporated into the empirical parameters of the extended Hückel Hamiltonian by fitting to ADC(2) excitation energies.

Table S4 of the Supporting Information summarizes the electronic structure of the heptazine and melem molecules, including their triplet states, highlighting the inverted singlet–triplet energy gap calculated with the ADC(2)­cc-pVDZ method. As reported in the literature, this singlet–triplet inversion in heptazine arises from the interplay between a larger contribution of double excitations in S_1_ compared to T_1_ (correlation effects) and a relatively small exchange energy.^8,9,55^ Consequently, the lowest singlet state lies at 2.70 eV, 0.25 eV below the first triplet state (*E*_T_1__ = 2.95 eV), in good agreement with previous theoretical calculations.^8,9,13,56^

Having demonstrated that our theoretical approach can accurately describe the excited states of heptazine in its planar ground-state geometry, we now present a comparison of the excited-state energies calculated using first principle methods and the e-Hückel model Hamiltonian. This comparison is based on geometry configurations of the heptazine molecule obtained from actual excited-state molecular dynamics simulations performed using the Ehrenfest-CSDM method. The comparison is shown in Figure 3.

**Figure 3:**
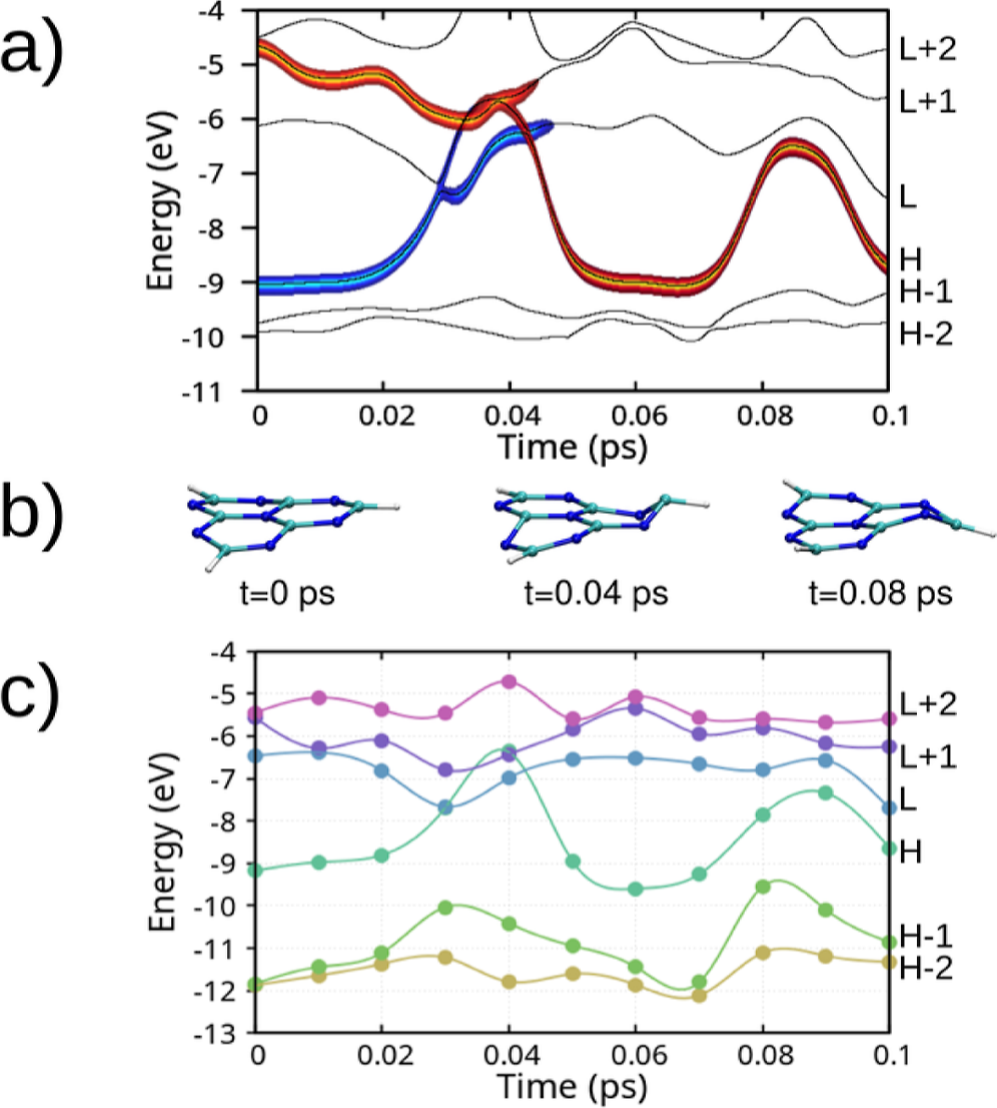
(a) Molecular orbital energies as a function of time for an actual excited-state molecular dynamics simulation of HTZ, performed using the Ehrenfest-CSDM method. The thick orange (blue) line indicates the occupation of the potential energy surfaces by the electron (hole) wave functions. (b) Snapshots of the heptazine molecule at *t* = 0, 0.04, and 0.08 ps. (c) Hartree–Fock single-point calculations for 11 molecular conformations equally spaced in time (dots), with the energy of the unoccupied molecular orbitals shifted downward to match the S_1_ excitation energy of 2.7 eV at *t* = 0 as obtained by the ADC(2) method. The solid lines are interpolation curves used for visualization purposes.

In Figure 3a, we present molecular orbital energies as a function of time for an excited-state molecular dynamics simulation initiated by the photoexcitation of the heptazine molecule to the S_5_ excited-state (as defined in the right side of Table 1). Figure 3b shows snapshots of the heptazine molecule at times *t* = 0, 0.04, and 0.08 ps of the nonadiabatic MD. For comparison with first principle calculations, Figure 3c presents Hartree–Fock single-point calculations for 11 molecular conformations equally spaced in time (dots), with the energy of the unoccupied molecular orbitals shifted down to match the S_1_ excitation energy of 2.7 eV at *t* = 0 as obtained by the ADC(2) method. The solid lines are interpolation curves used for visualization purposes. The correspondence between Figure 3a–c demonstrates that the transitions between frontier orbitals in the heptazine molecule adequately describe the single-particle excitations calculated using ADC(2) throughout the nonadiabatic molecular dynamics.

The alignment of the adiabatic potential energy surfaces matches well the observed Ehrenfest-CSDM exciton dynamics. Around 0.03 ps the system reaches a conical intersection, which results in negative ADC(2) excitations energies, an anticipated failure of the perturbative treatment. At 0.04 ps we observe an avoided crossing of S_1_ and S_2_ potential energy surfaces, which coincides with the transition of the electron from LUMO + 1 to LUMO orbital in the dynamics simulation, immediately followed by the relaxation to the ground state. At approximately 0.08 ps the system approaches a conical intersection again, while following the ground-state dynamics this time.

Having established that one-particle levels of the e-Hückel and shifted Fock Hamiltonians correspond well, we also analyze the ADC(2) potential energy surfaces along the simulated trajectory. While our nonadiabatic dynamics simulations focus entirely on the internal conversion dynamics, we acknowledge the importance of triplet states and the possibility of intersystem crossing. To get some preliminary qualitative insights, we calculate five lowest singlet and triplet excitation energies for each of the 11 configurations (Figure 4), with respect to the MP2 ground state energy.

**Figure 4:**
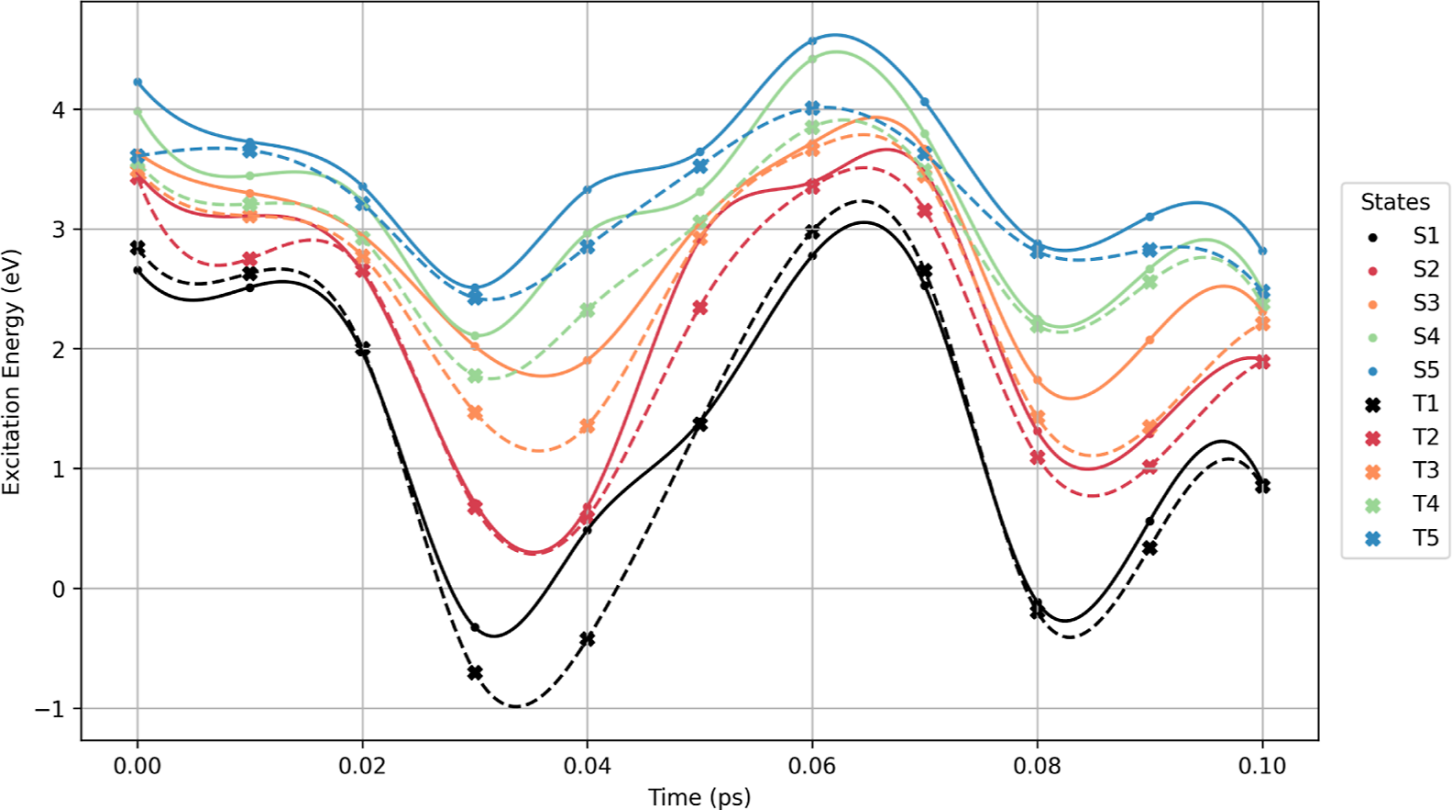
Five lowest singlet (circle, full line) and triplets (cross, dashed line) excitations energies calculated at the ADC(2)/cc-pVDZ level along the same molecular dynamics trajectory as in Figure 3. The MP2 ground state energy is the reference state for ADC(2) excitations energies.

At the initial configuration, the T_1_ state lies above S_1_ with a gap of approximately 0.18 eV, consistent with the value calculated at the optimized geometry. This reflects the unusual inverted singlet–triplet structure of HTZ, whereas higher-lying triplet states do not exhibit such inversion relative to their corresponding singlet states. During the dynamics, T_1_ crosses S_1_ multiple times, as shown in Figure 4, but the occurrences where T_1_ falls below S_1_ happen mostly in the vicinity of conical intersections, where the ADC(2) method is known to inadequately describe S_0_/S_*n*_ crossings. Nonetheless, the system exhibits favorable energetic conditions for intersystem crossing (ISC). Considering these limitations, we can infer the possible influence of intersystem crossing on the photophysical decay process. In heptazine, S_1_–T_1_ spin–orbit coupling is symmetry forbidden to first order, requiring spin-vibronic interaction for ISC to occur, particularly through the out-of-plane vibrational modes of heptazine.^10^ Consequently, periodic singlet–triplet crossings induced by out-of-plane vibrational modes, should induce continuous redistribution of the electronic population between the singlet and triplet states. This mechanism may prevent long-lived triplet trapping and could enhance photoluminescence efficiency, provided that internal conversion is suppressed.

We also analyzed the optimized geometry of the HTZ molecule in the excited state (Supporting Information, Section 2.2). In its ground state, the heptazine molecule exhibits a rigid planar structure with D_3*h*_ symmetry, as confirmed by our classical MD simulations. However, using the simulated annealing method to determine the excited-state geometry, we observe a symmetry reduction from D_3*h*_ to C_s_. In contrast, relaxation along the S_5_ state proceeds monotonically, accompanied by an additional out-of-plane displacement of a bridging aromatic carbon atom. In Figure S3, we compare the excitation energies of the S_1_ and S_5_ states of the heptazine molecule, calculated both with the semiempirical model, eq 6, and the ADC(2) method, for molecular geometries sampled along the adiabatic relaxation trajectories.

## 3 Results and Discussion

### 3.1 Heptazine Molecule

We now examine the excited-state dynamics of the heptazine molecule. The system was first prepared by thermalizing the molecule in the gas phase at *T* = 300 K for 200 ps. Following thermalization, 100 molecular configurations and velocities were generated at intervals of 3 ps to serve as initial conditions for the excited-state molecular dynamics simulations.

We assumed that vertical photoexcitations from the electronic ground state occur via the instantaneous absorption of a photon by the HTZ molecule. Two types of photoexcitations were considered: the S_0_ → S_1_ (HOMO–LUMO) transition and the S_0_ → S_5_ (HOMO–LUMO + 1) transition, as described in Table 1.

The S_1_ photoexcitation occurs in the visible range, with an energy of approximately 2.7 eV in acetonitrile.^57,58^ According to ADC(2) calculations (see Table 1), the S_0_ → S_1_ photoexcitation is symmetry-forbidden under *D*_3*h*_ symmetry in the dipole approximation. However, molecular vibrations and environmental interactions (e.g., with solvent) relax this selection rule, allowing for a residual oscillator strength for this transition.^57^ Consequently, experimental data on the excited state of the parent heptazine molecule is scarce, particularly concerning the excitation energy of the S_1_ state.

Considering the S_0_ → S_1_ excitation of the HTZ molecule and the subsequent excited-state nonadiabatic molecular dynamics, Figure 5 presents the behavior of the excitation energy across 100 independent molecular trajectories (gray) along with their averaged trajectory (blue). The decay curve of the individual trajectories are detailed in panels S5 and S6 of the Supporting Information.

**Figure 5:**
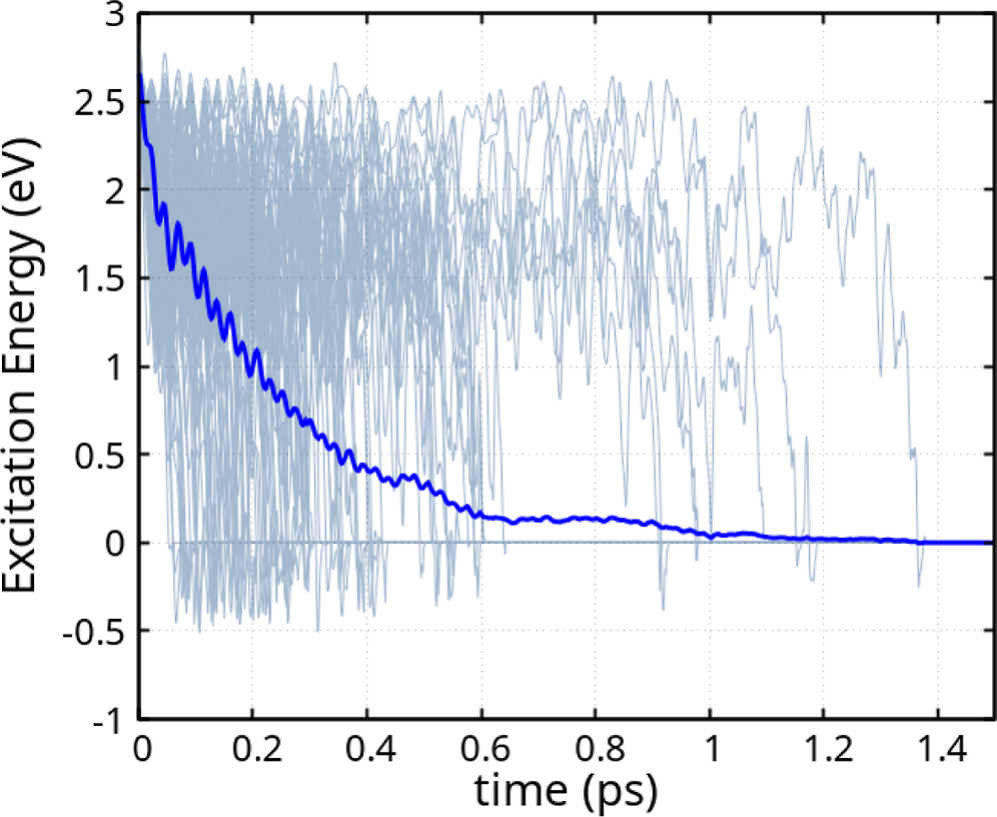
Excitation energy across 100 independent molecular trajectories (gray) and their averaged trajectory (blue), following the S_0_ → S_1_ excitation of the HTZ molecule and the subsequent excited-state nonadiabatic molecular dynamics.

Figure 6 describes a single trajectory of the excited-state dynamics following the S_0_ → S_1_ vertical photoexcitation of the heptazine molecule (trajectory #12, in panel S6), as calculated by the present Ehrenfest-CSDM approach. We observe that the energy of the excited-state (LUMO) stabilizes with the presence of the electron, while the energy of the ground-state becomes destabilized due to the hole in the HOMO. Two instances where the system approaches a conical intersection occur at *t* = 0.175 ps and *t* = 0.525 ps. However, only during the second encounter does the electron decay to the ground state, annihilating the hole, after which the energies of the LUMO and HOMO molecular orbitals resume oscillating around the equilibrium positions of these states. Additionally, we observe that the molecular distortion responsible for the internal energy conversion is the puckering of one of the rings, as shown by the insets.

**Figure 6:**
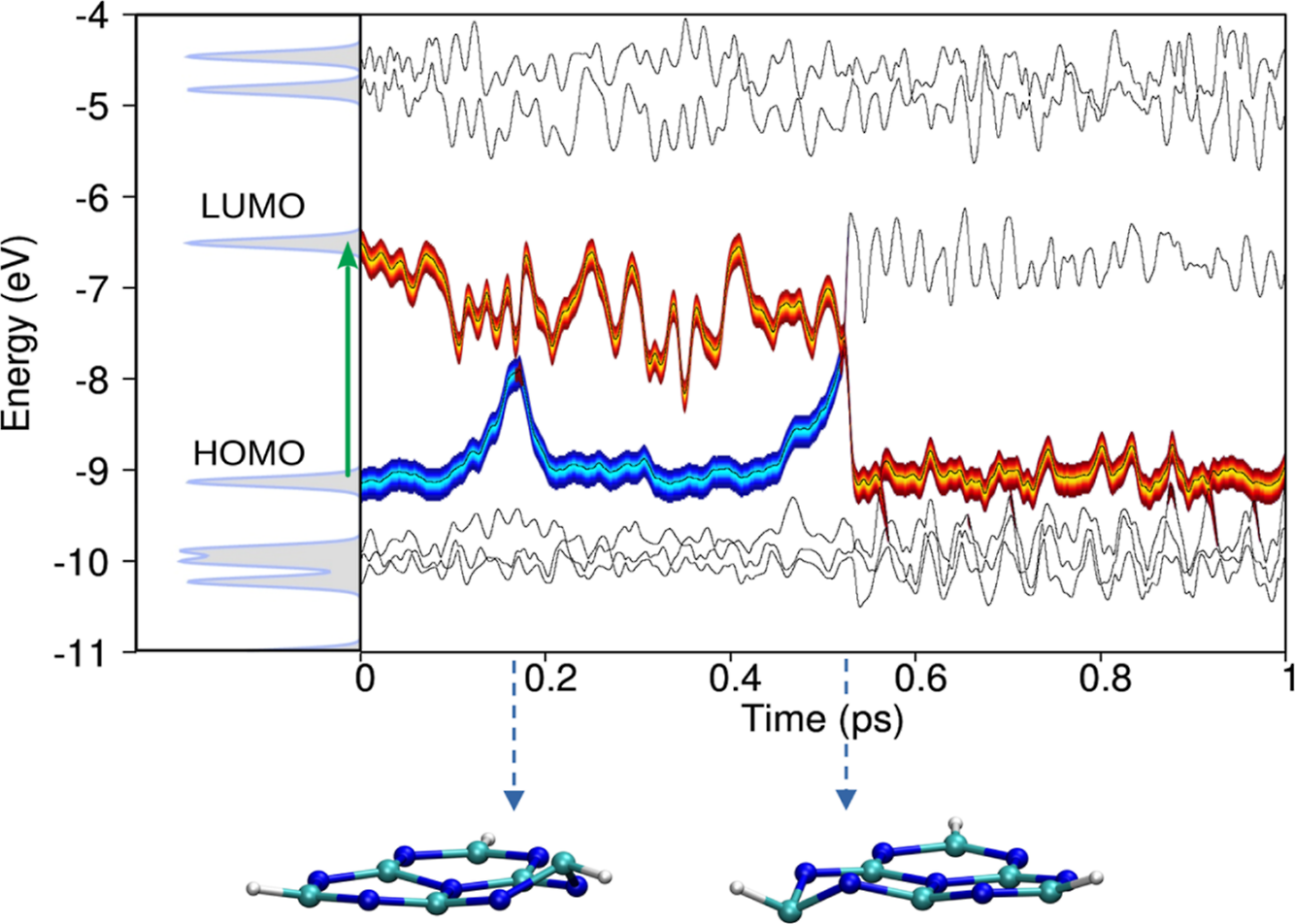
Dynamics of the photoexcited electron and hole pair in the photoexcited heptazine. On the left-hand side, the electronic density of states (DoS) of the HTZ molecule is shown. Molecular orbitals (MOs) are labeled, with a vertical green arrow denoting the S_1_ π → π* transition. The right-hand side displays MO energies (black lines) evolving over time, calculated dynamically during the excited-state nonadiabatic molecular dynamics (NAMD) simulation. Reference to the left panel allows tracking of the HTZ molecule’s MOs. The thick orange trace represents the electronic MO occupation, while the blue trace indicates the hole wave function occupation. At *t* = 0.525 ps, the blue trace merges with the orange, signifying electron–hole nonradiative recombination.

Theoretical calculations predict an energy of approximately 4.4 eV for the S_5_ π → π* photoexcitation of the heptazine molecule in the gas phase,^10,59^ along with a strong oscillator strength. Figure 7 presents excited-state molecular dynamics simulations following the S_0_ → S_5_ vertical photoexcitation for 100 initial configurations obtained after the thermalization of the system in the ground state (shown in gray). The figure also includes an average of the excitation energy over the independent trajectories. The dynamics of the individual 100 nonadiabatic trajectories are provided in the Supporting Information.

**Figure 7:**
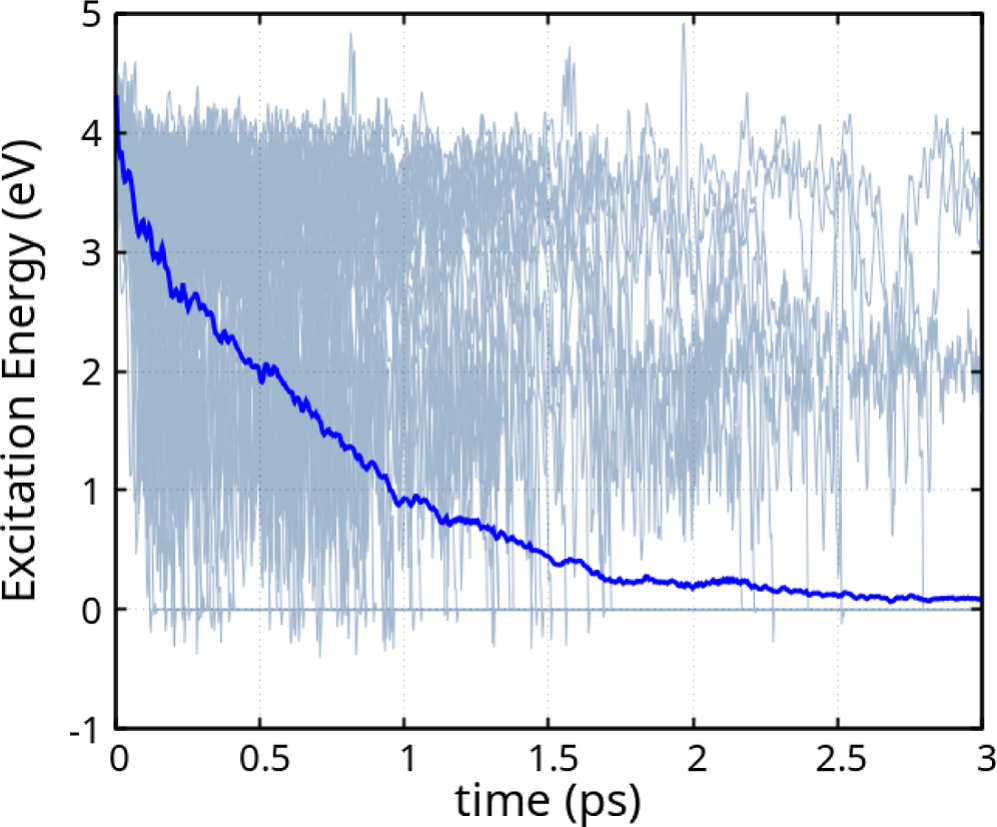
Excitation energy across 100 independent molecular trajectories (gray) and their averaged trajectory (blue), following the S_0_ → S_5_ excitation of the HTZ molecule and the subsequent excited-state nonadiabatic molecular dynamics.

In Figure 8, we analyze an individual nonadiabatic trajectory (trajectory #80, in panel S7) to understand the nonradiative relaxation mechanisms occurring in the heptazine molecule following the S_0_ → S_5_ excitation. The key points are summarized as follows:

1. The hole in the HOMO state induces puckering distortions in the carbon-nitride structure, significantly destabilizing the ground state of the heptazine molecule, as shown in the inset at *t* = 0.13 ps.
2. The electron in the [LUMO + 1] molecular orbital stabilizes this orbital relative to its quasi-degenerate counterpart. The lowest energy of [LUMO + 1] is reached when the bridge aromatic carbon is displaced out of the molecular plane, as illustrated in the inset at *t* = 0.6 ps.
3. At *t* ≈ 0.6 ps, the system decays from S_5_ → S_1_, where it remains until approximately *t* = 0.77 ps, at which point it undergoes the S_1_ → S_0_ transition.

**Figure 8:**
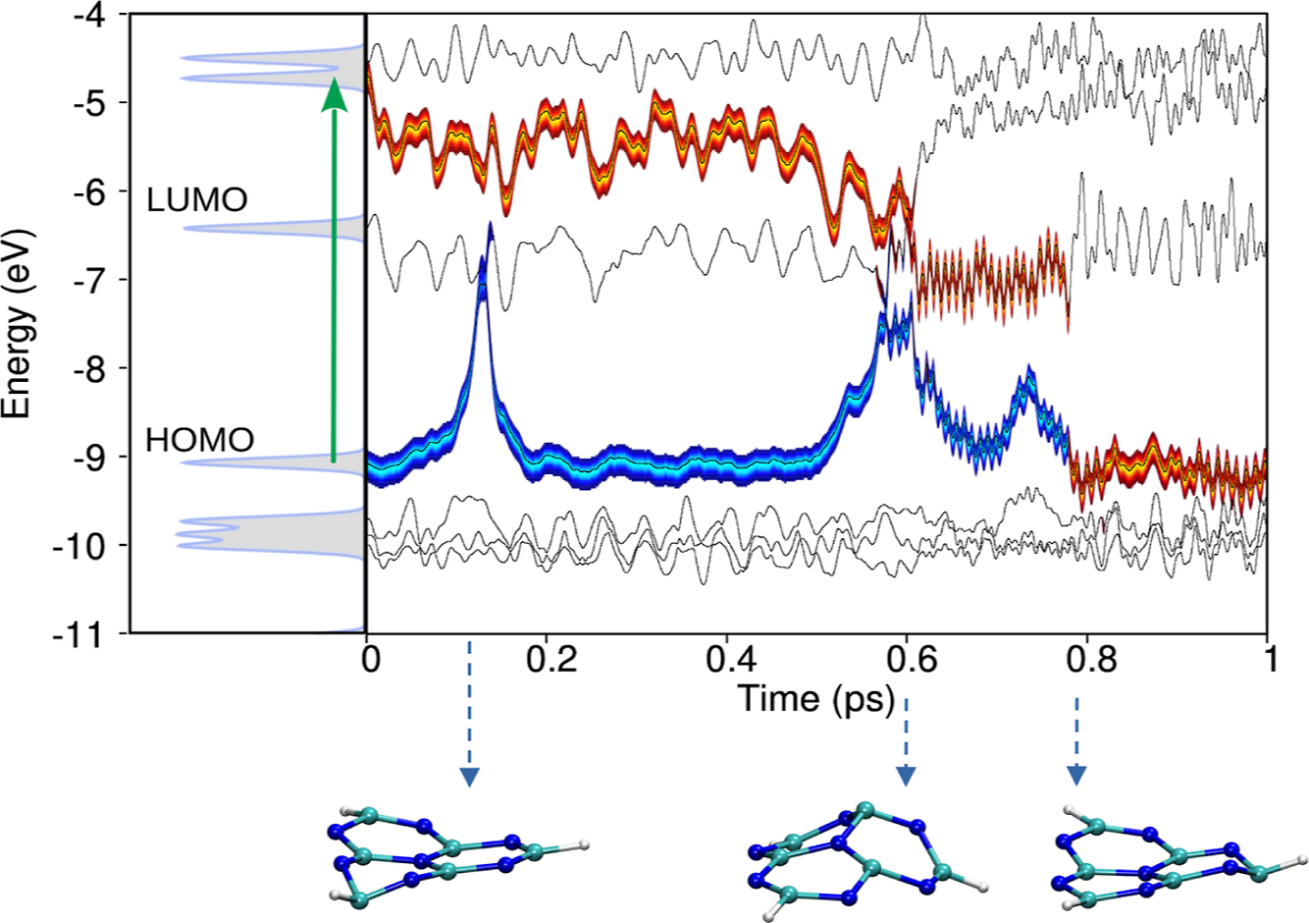
Dynamics of the photoexcited electron and hole pair in the photoexcited heptazine. On the left-hand side, the electronic density of states (DoS) of the HTZ molecule is shown. Molecular orbitals (MOs) are labeled, with a vertical green arrow denoting the S_5_ (*H* → *L* + 1) transition. The right-hand side displays MO energies (black lines) evolving over time, calculated dynamically during the excited-state nonadiabatic molecular dynamics (NAMD) simulation. Reference to the left panel allows tracking of the HTZ molecule’s MOs. The thick orange trace represents the electronic MO occupation, while the blue trace indicates the hole wave function occupation.

Regarding the Ehrenfest-CSDM approach, it is notable that electronic overcoherence is prevented throughout the excited-state dynamics. As molecular orbitals approach each other, coherence develops among them, but as they separate, this overcoherence is quickly quenched in favor of the pointer state, which drives the nonadiabatic dynamics. By analyzing individual trajectories, we conclude that the behavior of Ehrenfest-CSDM resembles that of the fewest-switches Surface-Hopping (FSSH) method, supporting the notion that Ehrenfest-CSDM successfully combines the strengths of Mean-field Ehrenfest and Surface Hopping into a single method without additional computational cost.

### 3.2 Melem Molecule

The frontier orbitals of MLM closely resemble those of heptazine, with an additional delocalization observed on the NH_2_ group in the LUMO. As MLM also belongs to the D_3*h*_ symmetry group, the S_0_ → S_1_ transition is symmetry-forbidden, resulting in a zero oscillator strength. In terms of excitation energies, the first singlet state (E_S_1__ = 3.82 eV) lies 0.36 eV below the first triplet state (E_T_1__ = 4.18 eV), consistent with previous calculations.^60^ Detailed information on the singlet and triplet electronic transitions, including excitation energies (calculated at the ADC(2)/cc-pVDZ level) and oscillator strengths (*f*), is provided in Table S4 of the Supporting Information.

Melem (MLM) is a compound with the molecular formula C_6_N_10_H_6_, derived from the heptazine (HTZ) molecule by substituting three hydrogen atoms with NH_2_ groups. Numerous studies have shown that nucleobases and their derivatives typically relax from the ^1^ππ* excited state to the ground state in under a picosecond, driven by ring puckering deformations.^21–26,29,30^ Notably, MLM’s chemical structure also closely resembles that of melamine (C_3_H_6_N_6_), for which transient absorption studies report an excited-state lifetime of approximately 13 ps.^27,61^ Interestingly, however, MLM exhibits high quantum-yield photoluminescence,^17,62^ which contrasts with the rapid nonradiative decay typically observed in structurally related molecules. Hereafter, we aim to clarify these observations and provide insights into the photorelaxation mechanisms of MLM compounds.

Quantum chemical studies suggest that derivatizing HTZ into MLM does not significantly alter the optimized ground-state structure of MLM.^5,8,63^ Accordingly, the same force field employed in previous HTZ simulations was used, with minor adjustments to account for the amino groups. To assess the accuracy of these parameters in describing MLM, we compared the optimized geometry results with those obtained using density functional theory (DFT) at the wB97xD/6-31g­(d,p) level of theory. As shown in Figure S1 (Supporting Information), the two approaches exhibit excellent agreement.

Although the structural parameters remain consistent, the excitation energies are modified, as detailed in Table S4 (Supporting Information). Table 2 summarizes the lowest singlet excitations considered in the dynamics simulations described below. To ensure consistency with the excitation energies of the melem molecule calculated using the ADC(2) method, for the part of this work that deals with the melem molecule, we designate the H → L + 1 excitation as the S_0_ → S_2_ transition.

**Table 2:** Electronic Transitions with Corresponding Excitation Energies and Oscillator Strengths (*f*) for the MLM Molecule in the Optimized Ground-State Geometry^a^

TURBOMOLE/ADC(2) cc-pVDZ				e-Hückel model
**transition**	*E* (eV)	*f*	MO transition	*E* (eV)
S_0_ → S_1_	3.82	0	H → L	3.82
S_0_ → S_2_	5.10	0.3424	H → L + 1	5.09
S_0_ → S_3_	5.10	0.3424	H → L + 2	5.10

a See Table S9 for comparison up to the S6 state.

System preparation followed the same procedure as for the HTZ molecule in the gas phase (3). Our simulations of the excited-state dynamics of the melem molecule, presented in Figure 9, depict the excitation energy as a function of time, for the molecule in the gas phase. Despite minor variations in the simulation framework, the excited-state lifetime of the melem molecule is considerably shorter than that of the heptazine molecule. We attribute this effect to the substitution of amino groups in place of the hydrogens in HTZ.

**Figure 9:**
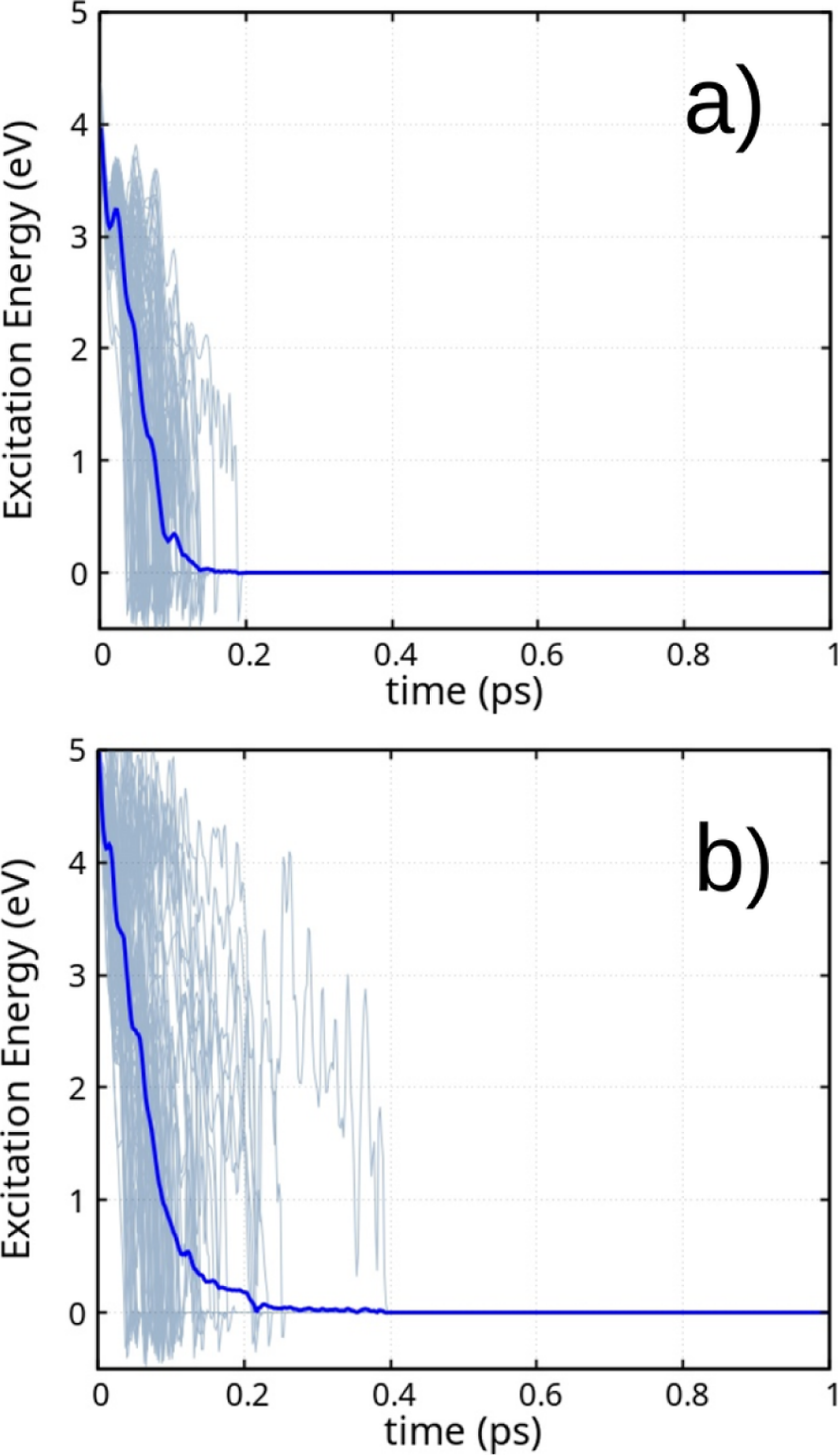
Excitation energy across 100 independent molecular trajectories (gray) and the corresponding averaged trajectory (blue), following the excitation of the melem molecule and the subsequent excited-state nonadiabatic molecular dynamics. Panel (a) shows the dynamics initiated from the S_0_ → S_1_ excitation, while panel (b) corresponds to the dynamics starting from the S_0_ → S_2_ excitation.

Amino and nitro substituent groups are well-known for modulating the fluorescence properties of organic dyes by introducing nonradiative energy dissipation pathways.^26,30–34,64^ Amino substitution, in particular, induces twisting and wagging deformations in the chromophore structure, promoting the formation of conical intersections with the ground state.^28,29^ Consequently, quenching rate constants can vary across several orders of magnitude, resulting in nonradiative decay time scales from a few hundred femtoseconds to nanoseconds.^64^ Furthermore, studies suggest that far-from-equilibrium conformations play a crucial role in nonradiative decay processes, which cannot be fully described by the simpler energy gap law.^34^ In Section 5.2 in the Supporting Information, we compare the internal conversion pathways of heptazine and melem, demonstrating that the MLM molecule experiences a stronger driving force from the Franck–Condon region toward the crossing point, resulting in a faster internal conversion.

### 3.3 Melem Aggregates

Here we examine the photorelaxation dynamics of melem aggregates in comparison with the isolated molecule. Large-scale excited-state nonadiabatic molecular dynamics simulations were performed on a six-molecule melem aggregate (6-MLM) in the gas phase, using the same simulation parameters as for the isolated molecule. Interesting structural features were observed as early as the system preparation phase. The initial setup involved six MLM molecules arranged in a planar configuration (Figure 10a), with each MLM making four hydrogen bonds, two with each nearest neighbor. This motif has been identified in STM images of self-assembled MLM structures on Au(111) surfaces.^65^

**Figure 10:**
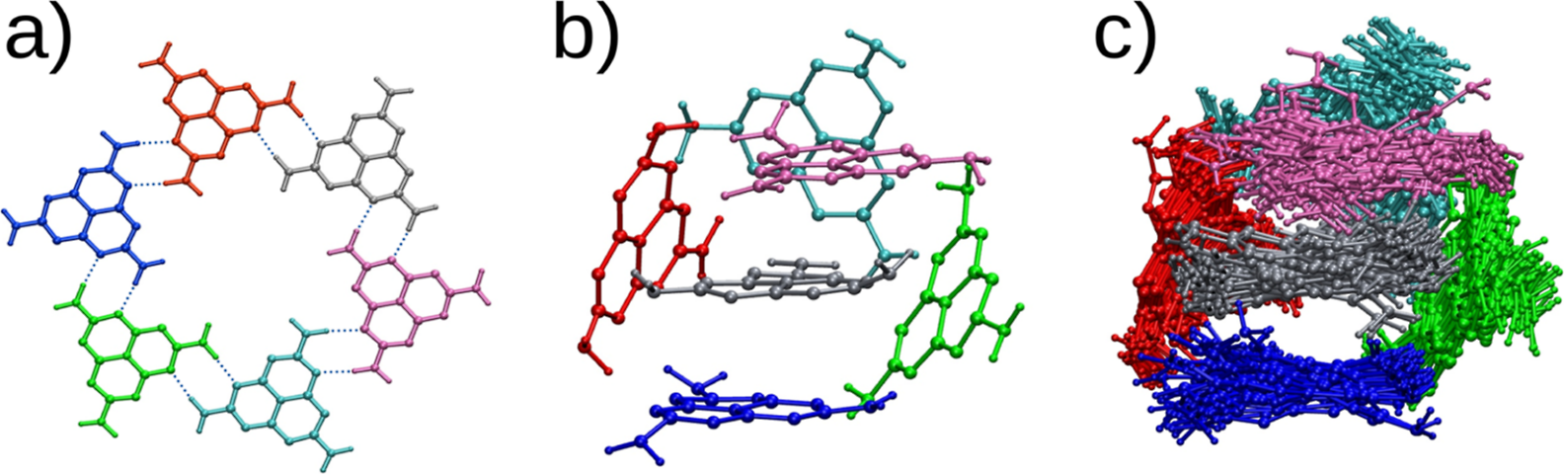
(a) Initial setup at *T* = 0 K, with six MLM molecules arranged in a planar configuration. (b) Structural transition of the 6-MLM aggregate from planar to three-dimensional during thermalization. (c) Fifty trajectory snapshots from a ground-state MD simulation at 300 K over 150 ps, depicting the structural evolution of the cluster.

During thermalization, the 6-MLM aggregate transitioned from a planar to a three-dimensional structure without dissociating even at temperatures of 500 K, demonstrating the robustness of the hydrogen bonds (Figure 10b). The three-dimensional arrangement increases the number of H-bonds per MLM molecule. Notably, each MLM molecule maintained its position within the aggregate, seldom exchanging positions with a neighboring molecule, thereby preserving the aggregate’s overall steric structure. Figure 10c presents a sequence of trajectory snapshots from a ground-state MD simulation at 300 K over 150 ps. Self-assembly of melem in a hot aqueous solution gives rise to hydrogen-bonded crystalline frameworks, where each MLM molecule is strongly attached to the neighboring molecules by multiple hydrogen bonds.^63,66^

The ground-state MD simulations at 300 K provided initial configurations for the excited-state nonadiabatic dynamics. For these simulations, we assumed that the MLM molecule located at the center of the aggregate (highlighted in gray in Figure 10c) was photoexcited at the start of the simulation; direct charge-transfer (CT) photoexcitations involving neighboring molecules were not considered. A representative excited-state nonadiabatic dynamics simulation is depicted in Figure 11. On the left-hand side of the figure, the electronic density of states (DoS) for the 6-MLM aggregate is displayed. The DoS projected onto the central MLM molecule is shown in gray, with its frontier molecular orbitals (MOs) labeled. A vertical green arrow marks the S_1_ π → π* transition localized on the central MLM molecule.

**Figure 11:**
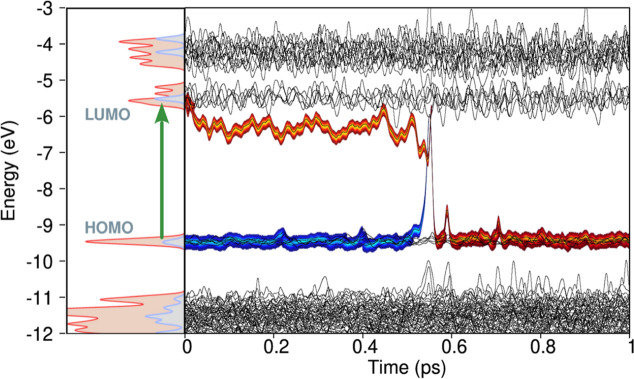
Dynamics of the photoexcited electron and hole pair in the 6-MLM aggregate. The vertical green arrow denotes the S_1_ π → π* transition in the central MLM molecule. The thick orange trace represents the electronic MO occupation, while the blue trace indicates the hole wave function occupation.

Figure 12 shows the time evolution of the excitation energy for 6-MLM aggregates across 50 independent excited-state nonadiabatic dynamics simulations, following S_0_ → S_1_ (a) and S_0_ → S_2_ (b) excitations. Detailed individual trajectories for both cases are provided in Figures S11 and S12. The results show significant suppression of photorelaxation in MLM-aggregates compared to isolated MLM molecules, shown in Figure 9. We attribute this outcome to two main factors: (1) inter-MLM charge transfer (of electrons and/or holes), which reduces the probability of electron–hole recombination via conical intersections, and (2) molecular packing, which restricts ring deformations, while H-bonds further constrain the twisting and wagging motions of the amino groups, reducing the occurrence of conical intersections.

**Figure 12:**
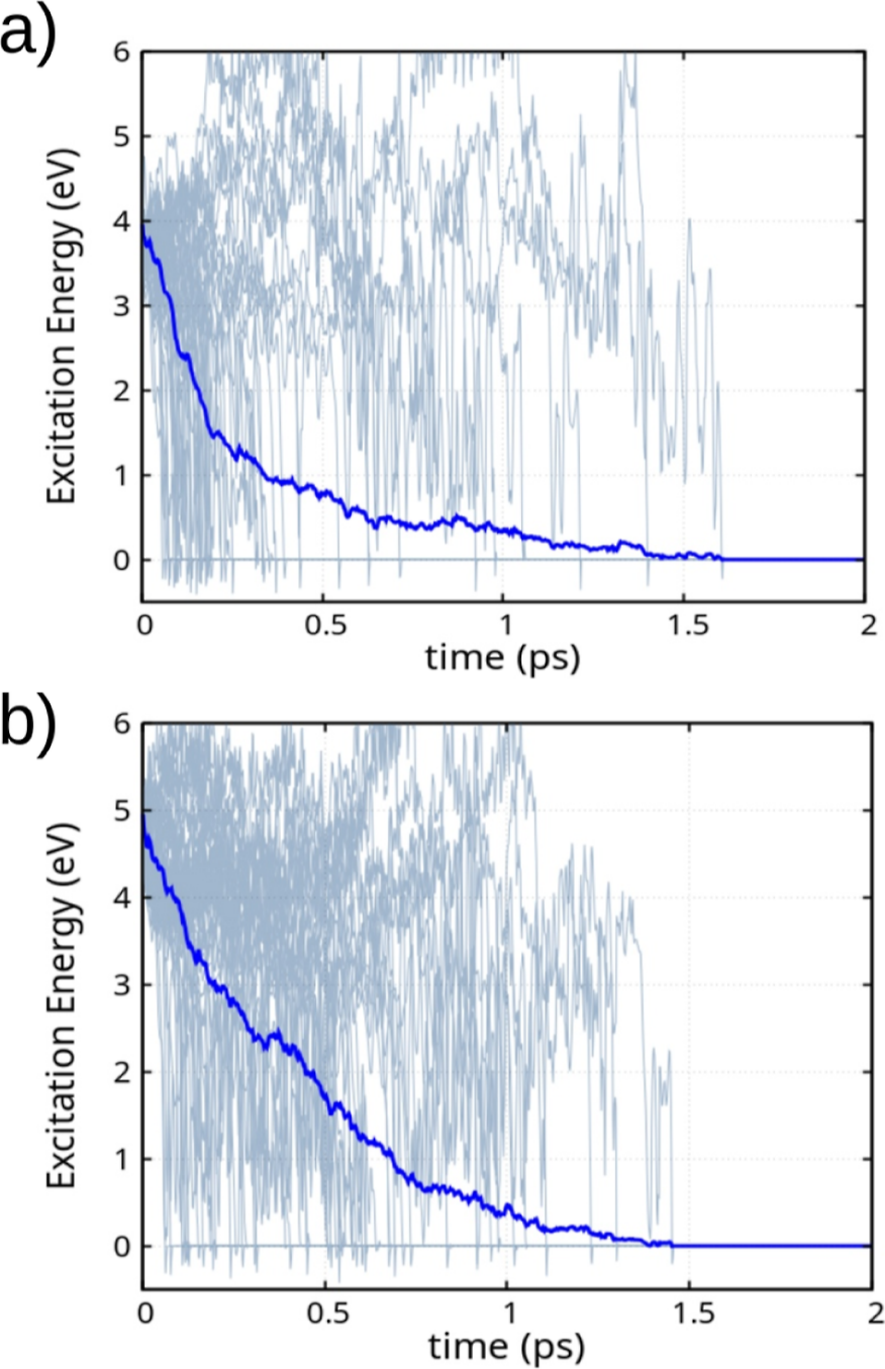
Excitation energy for 6-MLM aggregates across 50 independent molecular trajectories (gray) and the corresponding averaged trajectory (blue), following the excitation of the central MLM molecule within the aggregate and the subsequent excited-state nonadiabatic molecular dynamics. Panel (a) shows the dynamics initiated from the S_0_ → S_1_ excitation, while panel (b) corresponds to the dynamics starting from the S_0_ → S_2_ excitation.

Figure 13 illustrates the impact of inter-MLM charge transfer on electron–hole nonradiative recombination during the excited-state dynamics of the aggregate. For the 50 independent initial configurations analyzed in Figure 12, we tracked the dynamics of the photoexcited central MLM molecule (depicted in gray) until electron–hole recombination occurred via internal conversion. For the initial excitations S_0_ → S_1_ (a) and S_0_ → S_2_ (b), approximately 52% and 44% of the trajectories, respectively, result in recombination within the same MLM molecule that was initially excited (gray bar). In the remaining trajectories, recombination occurs in a different MLM molecule. The histogram bar colors correspond to the colors of the melem molecules shown in the inset of Figure 13c. Notably, nonradiative recombination of the electron and hole only occurs when they are located on the same MLM molecule. For larger clusters and more ordered crystals, we anticipate that the hopping process will further suppress nonradiative recombination.

**Figure 13:**
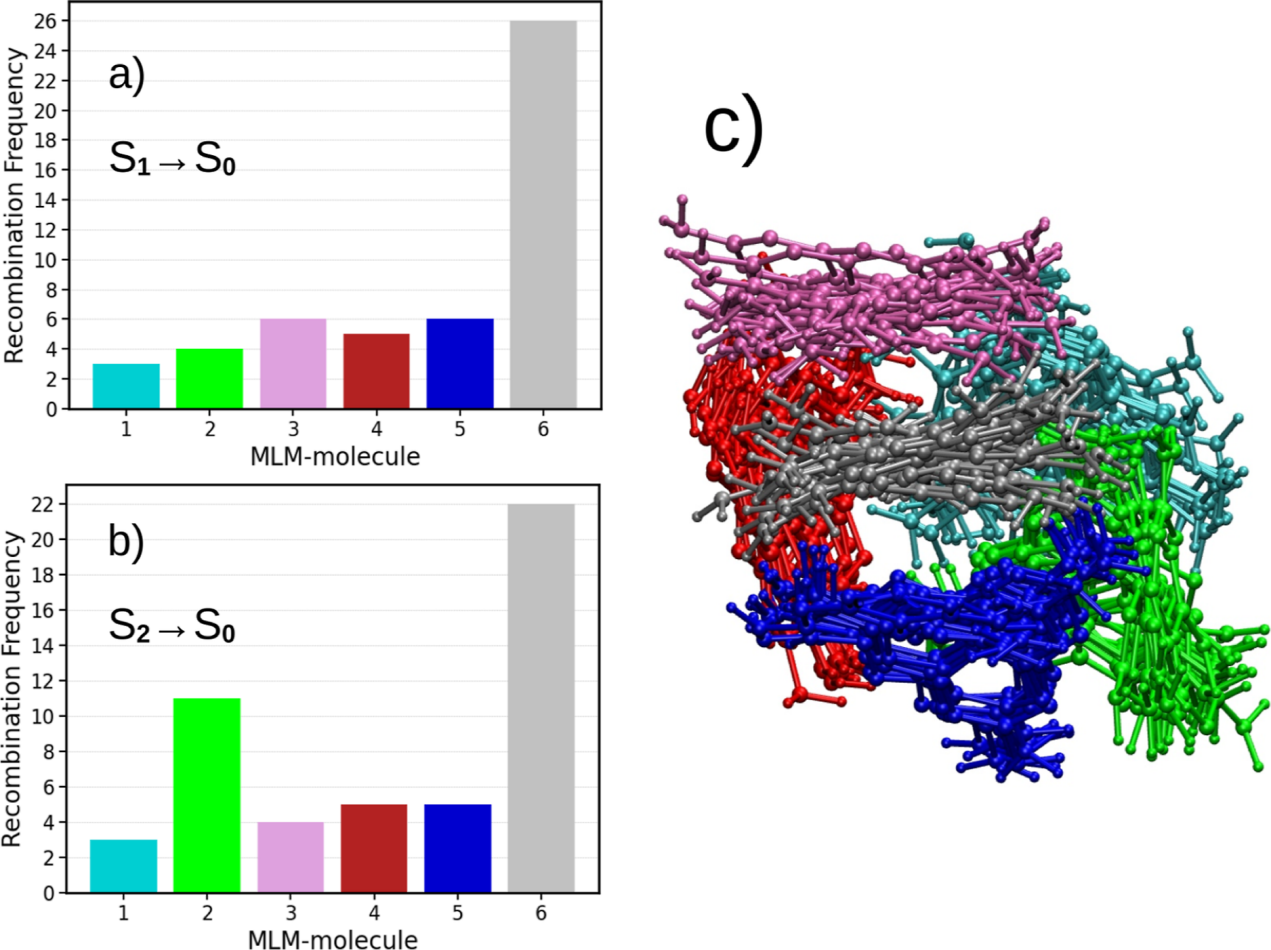
Histogram of electron–hole nonradiative recombination frequencies for S_0_ → S_1_ (a) and S_0_ → S_2_ (b) excitations. The colors of the histogram bars correspond to the melem (MLM) molecules depicted in the inset (c). At the start of the simulation, the MLM molecule at the center of the aggregate (shown in gray) was photoexcited.

To examine the effect of molecular packing on the mobility of melem, we analyzed 50 excited-state MD trajectories and compared the bending and twisting degrees of freedom of the molecule in both isolated and 6-MLM aggregate environments. Figure 14 shows the histogram of NH_2_ torsion angles for both cases. In the gas phase, the isolated melem molecule exhibits a broad distribution that is well described by a single Gaussian with a standard deviation of approximately σ ≈ 25°. In contrast, the torsional distribution in the aggregate is characterized by two Gaussian components in a 2:1 ratio, each with a narrower spread of σ ≈ 16°, indicating a significant restriction of the NH_2_ group’s twisting motion due to molecular packing. These results demonstrate that intermolecular interactions in the 6-MLM aggregate constrain the torsional mobility of the NH_2_ group, whereas the isolated molecule more readily adopts twisted geometries. Such twisted configurations are associated with the formation of twisted intramolecular charge transfer (TICT) states, which are known to efficiently undergo internal conversion (IC) to the ground state.^26,67^ Previous studies have shown that TICT states in amino-substituted molecules can be stabilized by hydrogen bonding in aqueous environments.^25,68^ In our case, similar stabilization occurs through strong intermolecular hydrogen bonds within the MLM aggregate, which both limit the formation of TICT states and suppress nonradiative decay via IC. A more detailed analysis is provided in Figure S14 of the Supporting Information.

**Figure 14:**
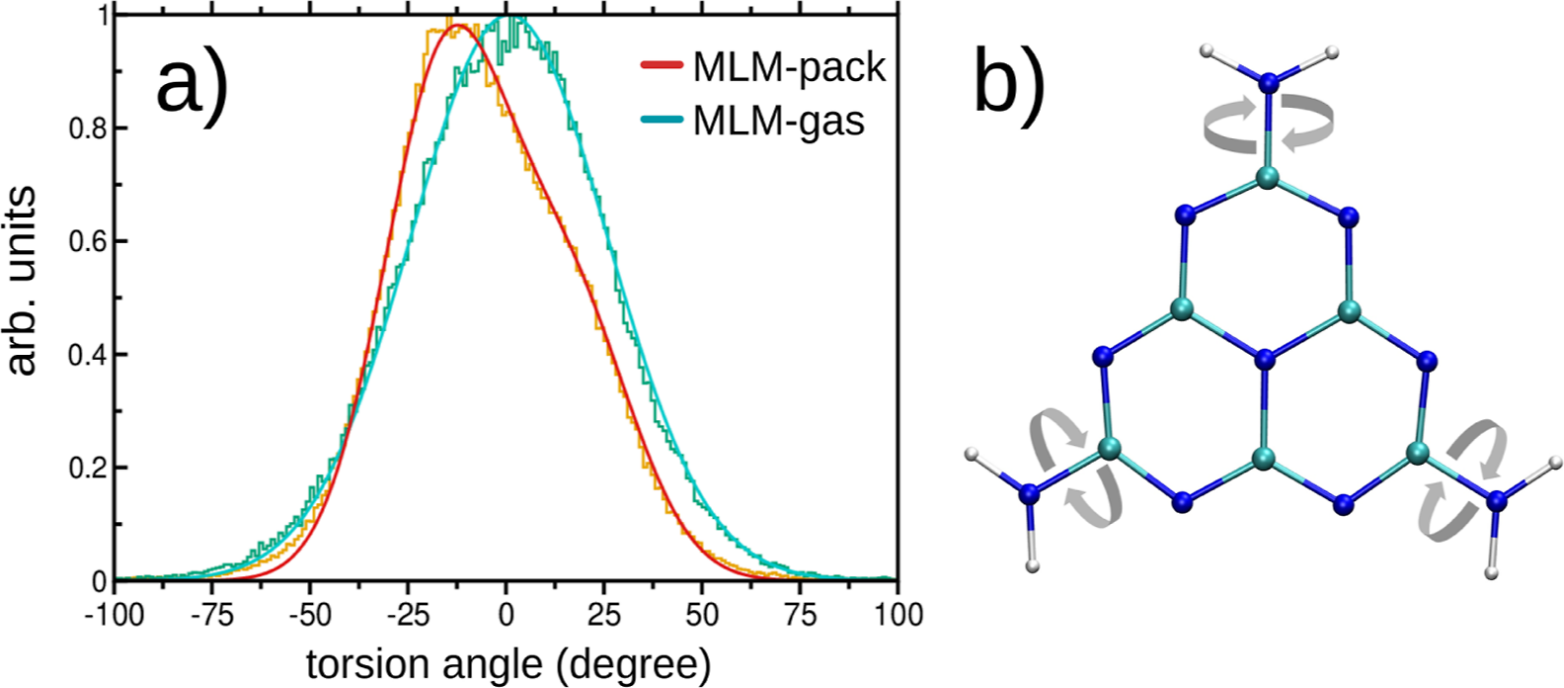
Effect of aggregation on molecular mobility. (a) Histogram of the torsion angles of the NH_2_ groups in the melem molecule in the gas phase (MLM-gas) and in the central MLM molecule within the 6-MLM aggregate (MLM-pack). (b) Illustration of the torsional degree of freedom.

The effect of molecular aggregation on the photophysics of heptazine-based systems can be inferred from studies on graphitic carbon nitride (g-C_3_N_4_). Nonadiabatic molecular dynamics simulations have been employed to investigate the dependence of recombination times on the size of the simulated supercell in 2D g-C_3_N_4_ monolayers.^69^ These studies report an increase in the recombination time scale from the picosecond regime in smaller supercells (2 × 2 system, containing 32 molecules) to the nanosecond regime in larger ones (10 × 10 system, containing 800 molecules). This behavior indicates that the longer diffusion length of electrons and holes in larger supercells suppresses geminate recombination. In addition, simulations on three-dimensional melon structures show that both electrons and holes can delocalize and transfer between adjacent heptazine units via π–π stacking interactions.^70^ This interunit transfer further enhances charge separation and contributes to the increase in photoluminescence quantum yield (PLQY) observed in larger supramolecular aggregates.

With regard to melem’s fluorescence in crystals and solutions,^17,66^ these findings indicate that, in addition to being a TADF material, melem’s high photoluminescence quantum yield is further enhanced by aggregation-induced emission (AIE). This conclusion aligns with melem’s low solubility in common solvents,^58,71^ which favors the formation of stable aggregates. In contrast, melamine, despite its similar chemical structure, exhibits significantly shorter fluorescence lifetimes,^61^ likely due to its higher solubility, which inhibits aggregation.

## 4 Conclusions

We have demonstrated the effectiveness of the semiempirical Ehrenfest method with Coherent Switches with Decay of Mixing (CSDM), highlighting its potential as a powerful tool for investigating the excited-state dynamics of large-scale molecular systems.

The present simulations highlight the critical role of nonsymmetric molecular vibrations in facilitating the nonradiative decay of photoexcited heptazine compounds. Our findings suggest that, in the gas phase, internal conversion is a major recombination channel, despite the thermal stability of the heptazine molecule. Intersystem crossing, not taken into account in our theoretical model, is also a relevant relaxation process that is enhanced by vibronic effects.^10,72–74^

Substitution of NH_2_ groups on the HTZ molecule to form melem decreases the excited-state lifetime of the latter due to the destabilizing effect of the amino groups, which promotes the formation of conical intersections with the ground state. This behavior is consistent with analogous aromatic amino compounds that exhibit lifetimes within the ps time scale range. However, it contrasts with the observation of MLM’s fluorescence in crystals and solutions. To address this issue, we investigated the photorelaxation dynamics of melem aggregates (6-MLM). Molecular dynamics simulations show the high stability of these aggregates, even at temperatures up to 500 K. Excited-state dynamics reveal that aggregation significantly suppresses relaxation mechanisms due to (i) inter-MLM charge transfer, which reduces the likelihood of electron–hole recombination via internal conversion, and (ii) molecular packing, which prevents ring deformation, thus reducing the occurrence of conical intersections. Our findings suggest that melem’s fluorescence is more likely produced by large aggregates rather than by individual molecules.

In a broader context of the efforts to design photoactive functional materials based on inverted singlet–triplet molecules, our results emphasize the necessity of accounting for the aggregation effects. As the radiative relaxation from S_1_ is intrinsically slow, the dynamics of internal conversion is critical. Here, we have demonstrated that the unfavorable trends based on single-molecule behavior can be reversed due to the intermolecular interactions in condensed phases. This observation is promising for the design of heptazine-based photocatalysts and light emitters, where for the latter the suppression of the internal conversion should be considered together with the enhancement of the oscillator strengths.

## Supplementary Material



## Notes

Includes: (1) The CSDM (Coherent Switch Decay of Mixing) Method, (2) Molecular Mechanics: Structures and Force Field Parameters, (3) Extended Hückel Parameters, and (4) excited-state nonadiabatic trajectories used in this study (PDF)
