# Supplementary material for: Aggregation Induced Effects on the Nonradiative Recombination Dynamics of Inverted Singlet–Triplet Heptazine-Based Materials
Source: J Phys Chem A. 2025 Jun 4;129(24):5220–33. doi: 10.1021/acs.jpca.5c00709 (PMC12186626; doi:10.1021/acs.jpca.5c00709)
Supplement: Supplementary file 1 [file jp5c00709_si_001.pdf]

Supporting Information for: Aggregation Induced Effects on the Nonradiative Recombination Dynamics of Inverted Singlet-Triplet Heptazine-Based Materials

Laure de Thieulloy,[†] Robson S. Oliboni,[‡] Piotr de Silva,[†] and Luis G. C. Rego[*,¶]

†*Department of Energy Conversion and Storage, Technical University of Denmark, 2800 Kongens Lyngby, Denmark*

‡*Department of Chemistry, Universidade Federal de Pelotas, Pelotas, RS, 96010-900, Brazil*

¶*Department of Physics, Universidade Federal de Santa Catarina, SC, 88040-900, Brazil*

E-mail: luis.guilherme@ufsc.br

1 The CSDM (Coherent Switch Decay of Mixing) Method

The hybrid quantum-classical hamiltonian of the molecular system in the Dynemol method is comprised of electrons and nuclei terms, the later also comprised of a classical force-field (FF) and an electronic quantum component ($\mathbf{H}^{eh}$)

$$\mathbf{H} = T + V = T + \left\{ V^{FF} + \langle \Psi | \mathbf{H}^{eh} | \Psi \rangle \right\} = T + V^{FF} + V^{QM} \tag{1}$$

with $|\Psi\rangle = \sum_i C_i |\phi_i\rangle$, $\{\phi\}$ is the adiabatic basis of $\mathbf{H}^{eh}$ and $V^{QM} = Tr\left[\rho \mathbf{H}^{eh}\right]$.

The electronic dynamics of the CSDM method is defined so that

$$\dot{\rho} = \dot{\rho}_{Coh} + \dot{\rho}_{Dech} \tag{2}$$

$$= \dot{\rho}_{TDSE} + \dot{\rho}_{Dech} \tag{3}$$

The time evolution of the actual density matrix of the electronic degrees of freedom is produced by both a coherent propagation, determined by the TDSE, and a decoherent part. Notice that, although $\dot{\rho}$ is simply defined as a sum of two contributions, the actual ρ matrix, and the wavepacket for that matter, is not given by a sum of two independent terms. So we will take advantege of $\dot{\rho}$.

Let us consider the conservation of energy for the hybrid hamiltonian. Due to overall energy conservation, we must have

$$\dot{\mathbf{H}} = \dot{T} + \dot{V} = 0 \ , \tag{4}$$

where the classical kinetic energy of the nuclei and the effective potential are given by

$$T = \sum_N \frac{\vec{P}_N \cdot \vec{P}_N}{2M_N} \Longrightarrow \dot{T} = \sum_N \frac{\vec{P}_N}{M_N} \cdot \dot{\vec{P}}_N = \sum_N \vec{v}_N \cdot \dot{\vec{P}}_N \tag{5}$$

$$V = V^{FF} + Tr\left[\rho \mathbf{H}^{eh}\right] \Longrightarrow \dot{V} = \dot{V}^{FF} + \dot{V}\big|_{Coh} + \dot{V}\big|_{Dech} \tag{6}$$

Considering the $\dot{V}$ term in the **adiabatic** representation, we get

$$\dot{V}^{QM} = \dot{V}\big|_{Coh} + \dot{V}\big|_{Dech} = \underbrace{\sum_i \dot{\rho}_{ii}\big|_{Coh} E_i + \sum_i \rho_{ii} \dot{E}_i}_{\dot{V}|_{Coh}} + \underbrace{\sum_i \dot{\rho}_{ii}\big|_{Dech} E_i}_{\dot{V}|_{Dech}} \tag{7}$$

Back to Eq. (8) we have

$$\dot{\mathbf{H}} = \left\{ \sum_N \vec{v}_N \cdot \dot{\vec{P}}_N\big|_{Coh} + \dot{V}^{FF} + \dot{V}\big|_{Coh} \right\} + \left\{ \sum_N \vec{v}_N \cdot \dot{\vec{P}}_N\big|_{Dech} + \dot{V}\big|_{Dech} \right\} = 0 \ . \quad (8)$$

To satisfy the equation above, both terms must vanish. Let us consider the coherent part first, as given in the present semiempirical approach:

$$\sum_N \vec{v}_N \cdot \dot{\vec{P}}_N\big|_{Coh} + \dot{V}^{FF} + \dot{V}\big|_{Coh} = 0 \quad (9)$$

with

$$\dot{V}\big|_{Coh} = \sum_i \dot{\rho}_{ii}\big|_{Coh} E_i + \sum_i \rho_{ii} \dot{E}_i \ , \quad (10)$$

$$\dot{\rho}_{ii}\big|_{Coh} = [\dot{C}_i^*]_{Coh} C_i + C_i^* [\dot{C}_i]_{Coh} = -\sum_{i \neq j} 2\mathbb{R}\left[\rho_{ij}\right] \sum_N \vec{v}_N \cdot \vec{d}_{ij}^N \quad (11)$$

$$\dot{E}_i = \sum_N \vec{v}_N \cdot \nabla_N E_i \quad (12)$$

Substituting Eqs. (11) and (12) into Eq. (10) gives

$$\dot{V}\big|_{Coh} = \sum_i \rho_{ii} \dot{E}_i + \sum_i \dot{\rho}_{ii}\big|_{Coh} E_i \quad (13)$$

$$= \sum_N \vec{v}_N \cdot \left\{ \rho_{ii} \nabla_N E_i - \sum_{i \neq j} 2\mathbb{R}\left[\rho_{ij}\right] E_i \vec{d}_{ij}^N \right\} \quad (14)$$

$$= \sum_N \vec{v}_N \cdot \nabla_N V^{QM} \ , \quad (15)$$

where

$$\vec{F}^{Ehrenfest} = -\nabla_N V^{QM} = -\rho_{ii} \nabla_N E_i + \sum_{i \neq j} 2\mathbb{R}\left[\rho_{ij}\right] E_i \vec{d}_{ij}^N \quad (16)$$

depends on the actual (CSDM) density matrix ρ, that also gives us $V^{QM} = Tr\left[\rho \mathbf{H}^{eh}\right]$.

Thus, Eq. (9) can be rewritten as

$$\sum_N \vec{v}_N \cdot \left\{ \vec{F}_{_N}\big|_{Coh} + \nabla_{_N} V^{FF} + \nabla_{_N} V^{QM} \right\} = 0 \tag{17}$$

which gives the coherent force on atom N

$$\vec{F}_{_N}\big|_{Coh} = -\nabla_{_N} V^{FF} - \nabla_{_N} V^{QM} \ , \tag{18}$$

that provides energy conservation in the coherent (Ehrenfest) part of the propagation.

In the CSDM method the total energy conservation (Eq. 8) is attained if the decoherent part of the propagation satisfies

$$\sum_N \vec{v}_N \cdot \dot{\vec{P}}_N\big|_{Dech} + \dot{V}\big|_{Dech} = 0 \ . \tag{19}$$

Assuming the first-order linear decay of coherence, we have

$$\dot{\rho}_{_{ii}}\big|_{Dech} = -\frac{\rho_{_{ii}}}{\tau_{ik}} < 0 \ , \quad i \neq k \tag{20}$$

$$\dot{\rho}_{_{kk}}\big|_{Dech} = \sum_{j \neq k} \frac{\rho_{_{jj}}}{\tau_{jk}} > 0 \ , \tag{21}$$

where i represents an arbitrary adiabatic state and k is the adiabatic **pointer-state**. Consequentely, the occupation of the pointer-state increases monotonically over time, at the expense of the occupations of the other states. Thrular and collaborators[1] derived the simplest function that satisfy the two main constraints: (i) energy conservation along the nonadiabatic compling vector direction, and (ii) the electronic decoherence (demixing) time being shorter than the shortest electronic time scale. The expression obtained for the decoherence time is

$$\tau_{ik} = \frac{\hbar}{|E_i - E_k|} \left(1 + \frac{0.1 * Hartree}{E_{kin}} \right) \ , \tag{22}$$

where E_{kin} is the kinetic energy of the nuclei and 0.1 is a phenomenological parameter. It

has been noted that the value of this parameter does not significanlty affect the simulation results.[1]

From the viewpoint of the wavepacket, the decoherence time is $\tau_{wvpckt} = 2\tau_{ik}$. Since, for $i \neq k$ we have

$$C_i \longrightarrow C_i e^{-\Delta t/(2\tau_{ik})} \ , \tag{23}$$

thus, for $i \neq k$, that leads to

$$\begin{aligned} \overset{\bullet}{\rho}_{ii} &= \frac{d}{dt}\left[C_i^* C_i\right] = \frac{C_i^*}{dt}\Big|_{Dech} C_i + C_i^* \frac{C_i}{dt}\Big|_{Dech} && (24) \\ &= -\frac{C_i^* C_i}{2\tau_{ik}} - \frac{C_i^* C_i}{2\tau_{ik}} = -\frac{\rho_{ii}}{\tau_{ik}} \ . && (25) \end{aligned}$$

The same procedure can be applied to $i = k$. Therefore, we have for $\overset{\bullet}{V}\big|_{Dech}$

$$\begin{aligned} \overset{\bullet}{V}\Big|_{Dech} &= \sum_{i \neq k} \overset{\bullet}{\rho}_{ii}\Big|_{Dech} E_i + \overset{\bullet}{\rho}_{kk}\Big|_{Dech} E_k && (26) \\ &= -\sum_{i \neq k} \frac{\rho_{ii}}{\tau_{ik}} \left(E_i - E_k\right) \ . && (27) \end{aligned}$$

Back to Eq. (19), we have

$$\sum_N \vec{v}_N \cdot \overset{\bullet}{\vec{P}}_N\Big|_{Dech} = \sum_{i \neq k} \frac{\rho_{ii}}{\tau_{ik}} \left(E_i - E_k\right) \ . \tag{28}$$

This equation defines the decoherent force, ensuring that it conserves total energy. The decoherent force on atom N, considering contributions from both electron and hole wavepackets, is calculated as

$$\begin{aligned} \vec{F}_N^{Dech} &= \sum_{i \neq k} \left\{ \vec{f}_{ik^e}^{N} - \vec{f}_{ik^h}^{N} \right\} && (29) \\ &= \sum_{\alpha=e,h} \sum_{i \neq k_\alpha} \Theta_\alpha \, \frac{\rho_{ii}^\alpha}{\tau_{ik_\alpha}} \frac{\left(E_i - E_{k_\alpha}\right)}{\left(\sum_N \vec{v}_N \cdot \hat{s}_{ik_\alpha}^N\right)} \hat{s}_{ik_\alpha}^N \ , && (30) \end{aligned}$$

where $\Theta_\alpha = +1$ for the electron and $\Theta_\alpha = +1$ for the hole, k_α is the associated pointer state, which may differ for electron and hole wavepackets, and $\hat{s}^N_{ik_\alpha}$ is the direction of the decoherent force, as defined in the Support Information.

2 Molecular Mechanics Force Field Parameters

2.1 Structures

Figure S1 shows an excellent match between the geometries optimized by the classical force field and those obtained from DFT for the heptazine molecule, as well as for the monomer, dimer, and trimer of the melem compound

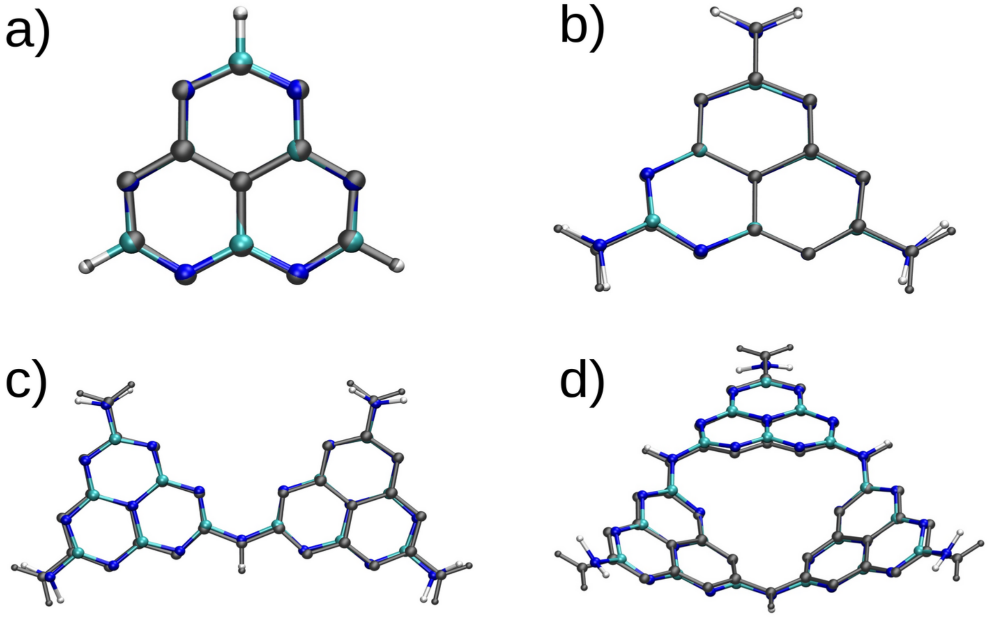

Figure S1: Comparison of optimized molecular structures based on ground-state energy obtained using the molecular mechanics framework (blue, cyan, and white atoms) and the DFT method (solid color) with the long-range corrected hybrid functional wB97xD and the 6-31g(d,p) basis set. Molecular structures shown are: (a) heptazine molecule, (b) melem molecule, (c) melem dimer, and (d) melem trimer.

In addition to accurately capturing the overall geometry, the classical force field successfully describes the two lowest-energy isomeric configurations of the MLM trimer, with

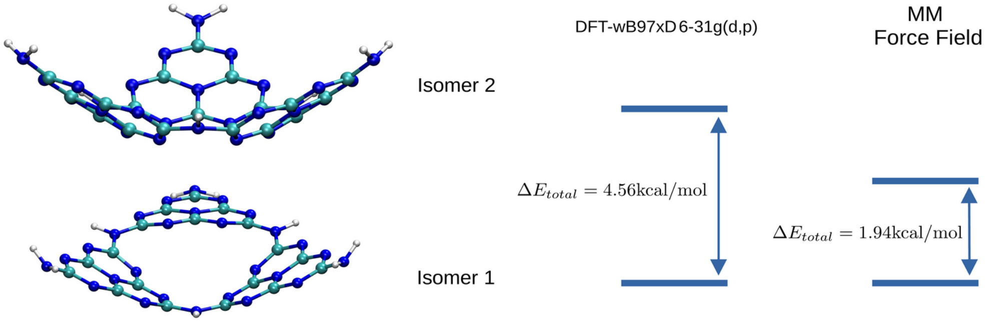

Figure S2: Lowest-energy isomers of the MLM trimer structure, with corresponding total energies calculated using the DFT and MM frameworks.

an energy deviation of approximately 2.5 kcal/mol relative to the DFT wB97xD/6-31g(d,p) calculations, as shown in Figure S2.

2.2 Excited-State Geometry

In its ground state, the heptazine molecule exhibits a rigid planar structure with D_{3h} symmetry, a characteristic confirmed by our classical MD simulations using the developed force field. However, when employing the simulated annealing method to determine the excited-state geometry, we observe a symmetry reduction from D_{3h} in the ground state to C_s in the excited state. Figure S3-a shows the semiempirical excitation energy,

$$V_{EH}\left[\Psi^{el}(\mathbf{R},t),\Psi^{hl}(\mathbf{R},t)\right] = \mathrm{Tr}\left[\rho^{EH}(\mathbf{R},t)\mathbf{H}(\mathbf{R}_t)\right], \tag{31}$$

as defined in Eq. (5) of the paper, for the heptazine molecule in the S_1 state along the adiabatic relaxation pathway. For comparison, we also include excitation energies calculated with the ADC(2) method for selected points along the trajectory. The simulated annealing in the S_1 state was initiated from the ground-state geometry and performed with a phonon relaxation time $\tau = 0.1$ fs to minimize heating of the nuclear structure, concluding at a final temperature of $T = 0.1$ K.

In the S_1 state, structural relaxation occurs in three stages. Initially, the atoms relax

within the molecular plane. Subsequently, the C–H bonds and the central N atom shift slightly out of the plane, preserving a C_{3v} symmetry. Finally, one of the C–H bonds undergoes further displacement, leading to a puckered distortion characterized by C_s symmetry (see inset of Figure S3-a). In the S_5 state (Figure S3-b), the relaxation is monotonic, featuring an additional out-of-plane displacement of a bridging aromatic carbon atom. The comparison between the semiempirical excitation energies and those obtained from ADC(2) calculations is also good.

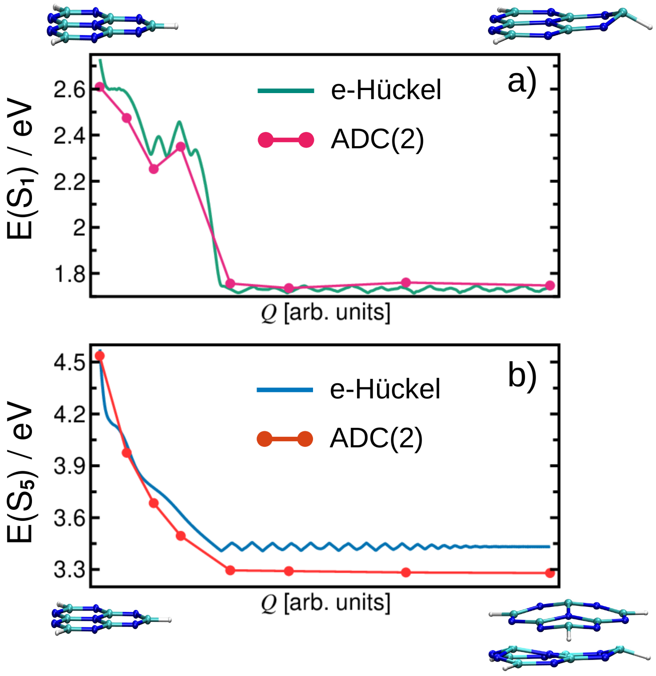

Figure S3: Excitation energies of the heptazine molecule calculated by the e-Hückel model and the ADC(2) method, for molecular geometries along the adiabatic relxation trajetories. The insets show the molecule's initial geometry (left) and its final geometry after stabilization in the excited-state (right).

2.3 Force Field Parameters

2.3.1 Heptazine Molecule

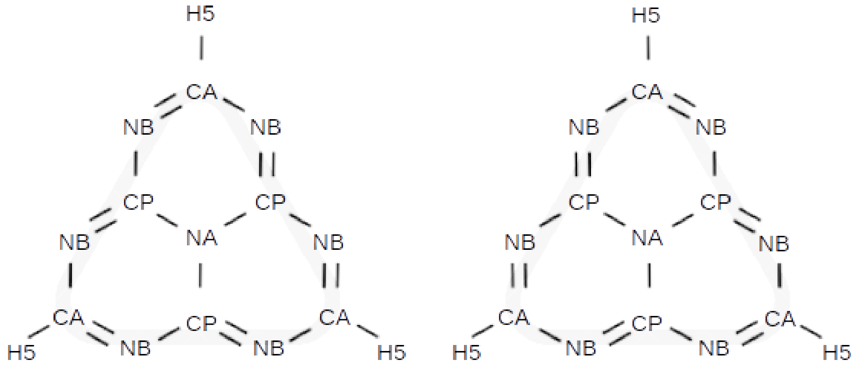

Figure S4: Atom types for the heptazine molecule, as defined by the GAFF2 force field model.[2] The gray contour highlights the external aromatic ring.

Table S1: Force Field parameters for the Heptazine (THZ) molecule, as defined by the GAFF2 (version 2.2.20) model.

Bond/Angle	Energy (kcal·mol^{-1}/Å^2)	Length (Å)	Angle (degrees)	Energy (kcal·mol^{-1})
CP-NA	296.53	1.4172	-	-
CP-NB	388.52	1.3377	-	-
CA-NB	386.49	1.3392	-	-
CA-H5	357.59	1.0880	-	-
CA-NB-CP	-	-	118.09	82.90
H5-CA-NB	-	-	115.79	50.16
NB-CP-NB	-	-	125.84	73.26
NB-CA-NB	-	-	127.30	72.76
CP-NA-CP	-	-	118.01	78.32
NA-CP-NB	-	-	114.96	74.32

torsion dihedral	N. of Paths	Energy (kcal/mol)	Phase (degrees)	Periodicity	Notes
X-CA-NB-X	2	9.60	180	2	same as X-CA-NC-X
X-CP-NA-X	4	9.30	180	2	from parm99 (X-CR-NA-X)
X-CP-NB-X	2	9.60	180	2	from GAFF2 (X-CA-NB-X)
	2	10.0	180	2	similar to parm99 (X-CR-NB-X)

Table S2: Force Field parameters for the Heptazine (THZ) molecule, as defined by the GAFF2 (version 2.2.20) model. Improper Dihedral: central atom in 3rd position

**improper dihedral**	**Energy (kcal/mol)**	**Phase (degrees)**	**Periodicity**	**Notes**
NB-NB-CA-H5	1.10	180.00	2	bsd.on C6H6 nmodes
CP-CP-NA-CP	1.10	180.00	2	from GAFF2 (CA-CA-NA-C3)
NA-NB-CP-NB	1.10	180.00	2	GAFF2 (NA-N2-CA-N2)
	1.10	180.00	2	parm99 (N2-NA-CA-NC)

Table S3: Force Field parameters for the Heptazine (THZ) molecule. AM1-BCC charges and Lennard-Jones parameters.

**Atom Type**	**Charge**	ε **(kcal/mol)**	σ **(Å)**
NA	-0.538500	0.170	3.648
CP	0.839500	0.086	3.816
NB	-0.737000	0.170	3.648
CA	0.734900	0.086	3.816
H5	0.078100	0.015	2.718

2.3.2 Melem Molecule

Table S4: Additional force field parameters for the Melem (MLM) molecule, defined by the GAFF2 model (version 2.2.20), provided to complement the HTZ parameter set.

**Bond**	**Energy ($kcal \cdot mol^{-1}/Å^2$)**	**Length (Å)**
NH-HN	529.50	1.0120
CA-NH	310.84	1.4030

**Angle**	**Energy (kcal/mol)**	**Angle (degrees)**
HN-NH-HN	39.50	115.12
NB-CA-NH	74.01	117.13
CA-NH-HN	48.80	116.07
CA-NH-CA	77.99	121.41

**torsion dihedral**	**N. of Paths**	**Energy (kcal/mol)**	**Phase (degrees)**	**Periodicity Periodicity**	**Notes**
X-CA-NH-X	4	9.60	180	2	from AMBER

**improper dihedral**	**Energy (kcal/mol)**	**Phase (degrees)**	**Periodicity**	**Notes**
CA-HN-NH-HN	1.10	180.00	2	from AMBER X-X-N2-H
CA-CA-NH-HN	10.0	180.00	2	to fit DFT calculations

**Atom**	**Antechamber Type**	**AMBER Charge (AM1-BCC)**
NA	na	-0.590454
CP	cc	0.884545
NB	nc	-0.817954
CA	cd	0.970945
NH	nh	-0.938454
NB	nd	-0.817954
HN	hn	0.457845

3 Electronic Excitation Energies of HTZ and MLM based on ADC(2) Calculations

For both, HTZ and MLM molecules, the first singlet and triplet excited states are both dominated by a HOMO-to-LUMO transition, corresponding to a $\pi \rightarrow \pi^*$ electronic excitation. The HOMO is predominantly localized on the peripheral nitrogens, whereas the LUMO is localized on the peripheral carbons and the central nitrogen of the molecule. The $S_0 \rightarrow S_1$ transition in the Franck–Condon region is symmetry-forbidden, resulting in an oscillator strength of zero.

As reported in the literature on the singlet–triplet inversion of the heptazine molecule, this phenomenon arises from the interplay between a larger contribution of double excitations to S_1 compared to T_1 (correlation effects) and a relatively small exchange energy.[3–5] Consequently, the lowest singlet state lies at 2.70 eV, 0.25 eV below the first triplet state ($E_{T_1} = 2.95$ eV), in good agreement with previous theoretical calculations.[3,4,6,7]

Table S5: Electronic transitions with corresponding excitation energies (ADC(2)/cc-pVDZ) and oscillator strengths (f) for HTZ and MLM molecules in the optimized ground-state geometry (DFT-wB97xD/6-31g(d,p))

	**HTZ**			**MLM**		
**Transition**	**E (eV)**	**f**	**MO Transition**	**E (eV)**	**f**	**MO Transition**
$S_0 \rightarrow S_1$	2.70	0	H → L	3.82	0	H → L
$S_0 \rightarrow S_2$	3.78	0	H-3 → L	5.10	0.3424	H → L+1
$S_0 \rightarrow S_3$	3.87	0	H-1 → L	5.10	0.3424	H → L+2
$S_0 \rightarrow S_4$	3.87	0	H-2 → L	5.58	0.541	H-1 → L
$S_0 \rightarrow S_5$	4.65	0.273	H → L+1	5.58	0.541	H-2 → L
$S_0 \rightarrow S_6$	4.65	0.273	H → L+2	6.03	0	H-3 → L
$S_0 \rightarrow T_1$	2.95	-	H → L	4.18	-	H → L
$S_0 \rightarrow T_2$	3.77	-	H-3 → L	4.41	-	H → L+2
$S_0 \rightarrow T_3$	3.84	-	H-1 → L	4.41	-	H → L+2
$S_0 \rightarrow T_4$	3.84	-	H-2 → L	5.08	-	H-1 → L
$S_0 \rightarrow T_5$	3.85	-	H → L+1	5.08	-	H-1 → L
$S_0 \rightarrow T_6$	3.85	-	H → L+2	5.17	-	H-3 → L

4 Extended-Hückel Parameters

4.1 Heptazine Molecule

Table S6: Optimized extended Hückel parameters for the heptazine molecule. Molecular orbital isosurfaces from [HOMO-3] to [LUMO+2] are shown in Figure 1 of the main text. The corresponding transition energies are listed in Table 1 of the main text.

Symbol	EHsymbol	residue	NoAt	Nvalen	n	spdf	IP	zeta	k_WH
N	NA	HTZ	7	5	2	s	-25.53577	2.199313	1.746490
N	NA	HTZ	7	5	2	p	-13.68473	2.199017	1.233170
C	CP	HTZ	6	4	2	s	-20.80815	2.039121	1.665337
C	CP	HTZ	6	4	2	p	-10.73872	2.213892	2.950006
N	NB	HTZ	7	5	2	s	-25.84915	1.872922	1.286840
N	NB	HTZ	7	5	2	p	-12.82828	1.807415	2.080830
C	CA	HTZ	6	4	2	s	-20.56135	2.301677	1.579987
C	CA	HTZ	6	4	2	p	-10.75076	2.230858	3.833606
H	H5	HTZ	1	1	1	s	-13.60000	1.300000	1.750000

Table S7: Electronic transitions with corresponding excitation energies and oscillator strengths (f) for the $C_6N_7H_3$ molecule in the optimized ground-state geometry.

**Turbomole / ADC(2) cc-pVDZ**			**e-Hückel model Hamiltonian**	
**Transition**	**E (eV)**	**f**	**MO Transition**	**E (eV)**
$S_0 \rightarrow S_1$	2.70	0	H → L	2.769
$S_0 \rightarrow S_2$	3.78	0	H-3 → L	3.698
$S_0 \rightarrow S_3$	3.87	0	H-1 → L	3.777
$S_0 \rightarrow S_4$	3.87	0	H-2 → L	3.778
$S_0 \rightarrow S_5$	4.65	0.273	H → L+1	4.656
$S_0 \rightarrow S_6$	4.65	0.273	H → L+2	4.661

4.1.1 Excited-States Dynamics

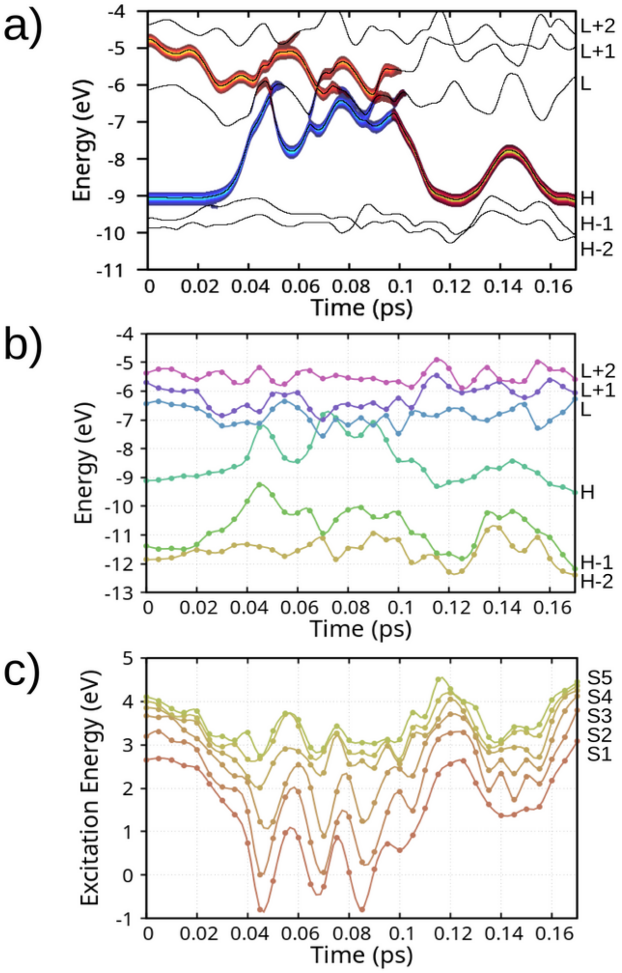

Figure S5: a) Molecular orbital energies as a function of time for an actual excited-state molecular dynamics simulation, performed using the Ehrenfest-CSDM method. The thick orange (blue) line indicates the occupation of the potential energy surfaces by the electron (hole) wavefunctions. b) Hartree-Fock single-point calculations for 11 molecular conformations equally spaced in time (dots), with the energy of the **unoccupied** molecular orbitals shifted downward to match the S_1 excitation energy of 2.7 eV at t=0 as obtained by the ADC(2) method.The solid lines are interpolation curves used for visualization purposes. c) ADC(2)cc-pVDZ excitation energies relative to the S_0 state.

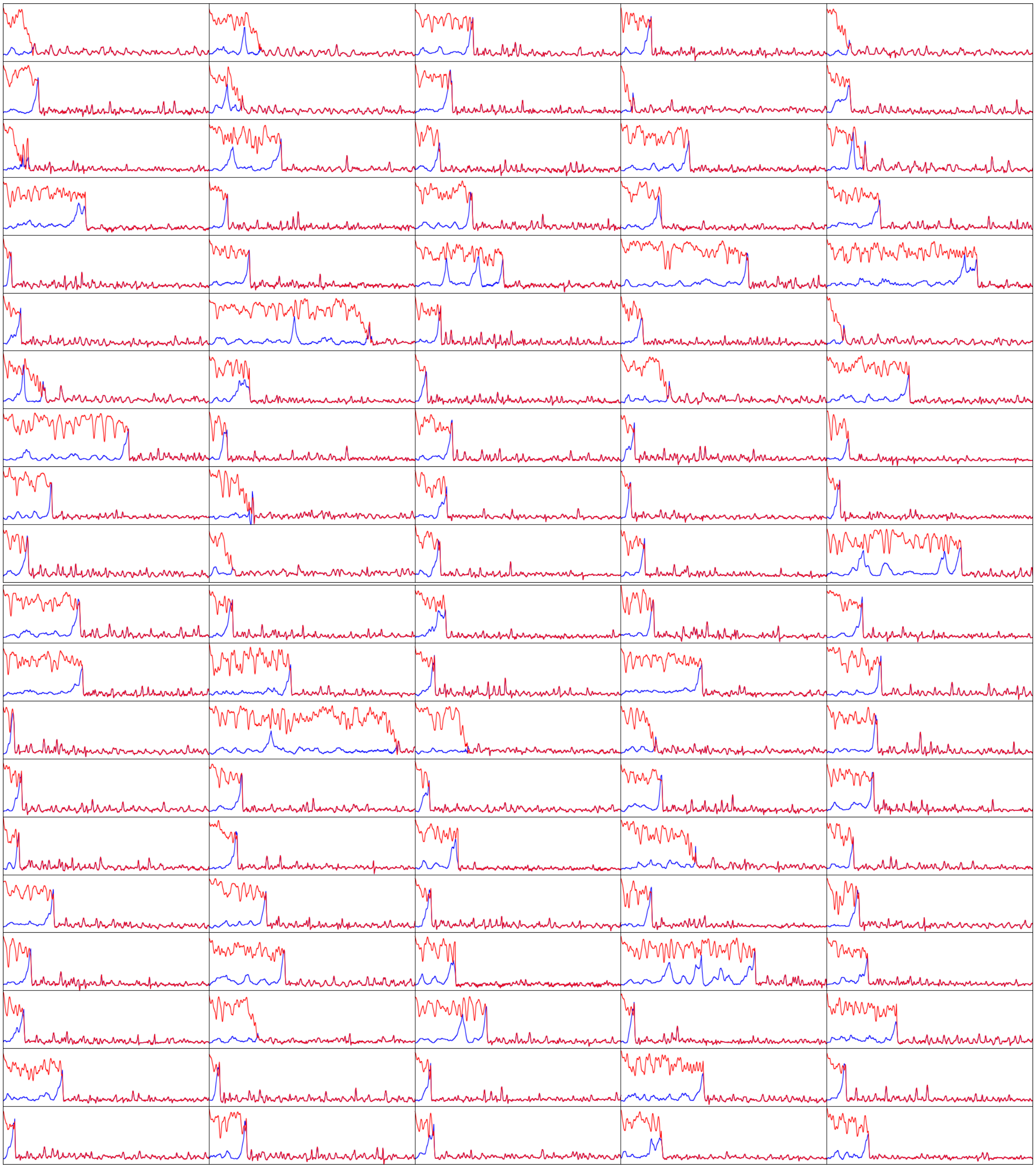

Figure S6: One hundred excited-state nonadiabatic molecular dynamics trajectories were generated following the $S_0 \to S_1$ excitation of the HTZ molecule. For all the graphs the vertical axis describes energy in the range $\varepsilon = [-9.5, -6.2]$ eV whereas the horizontal axis describes the time in the range $t = [0, 1.5]$ ps. The curves represent the expectation values of the energy, $\varepsilon_\alpha = \langle \psi_\alpha(t) | \hat{H} | \psi_\alpha(t) \rangle$, for the electron ($\alpha =$ el, red) and the hole ($\alpha =$ hl, blue).

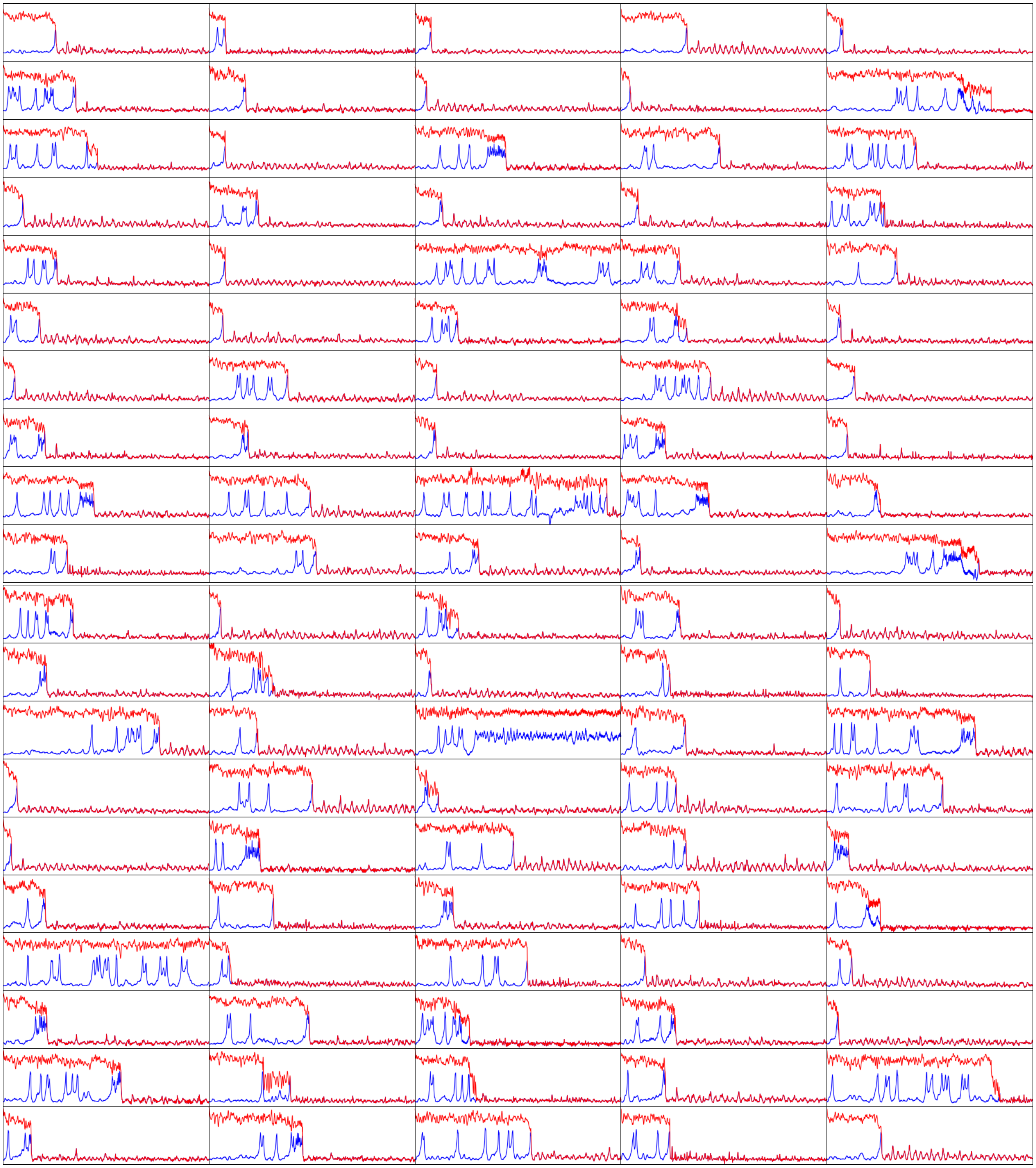

Figure S7: One hundred excited-state nonadiabatic molecular dynamics trajectories were generated following the $S_0 \to S_5$ excitation of the HTZ molecule. For all the graphs the vertical axis describes energy in the range $\varepsilon = [-10, -4.2]$ eV whereas the horizontal axis describes the time in the range $t = [0, 3]$ ps. The curves represent the expectation values of the energy, $\varepsilon_\alpha = \langle\psi_\alpha(t)|\hat{H}|\psi_\alpha(t)\rangle$, for the electron ($\alpha =$ el, red) and the hole ($\alpha =$ hl, blue).

4.2 Melem Molecule

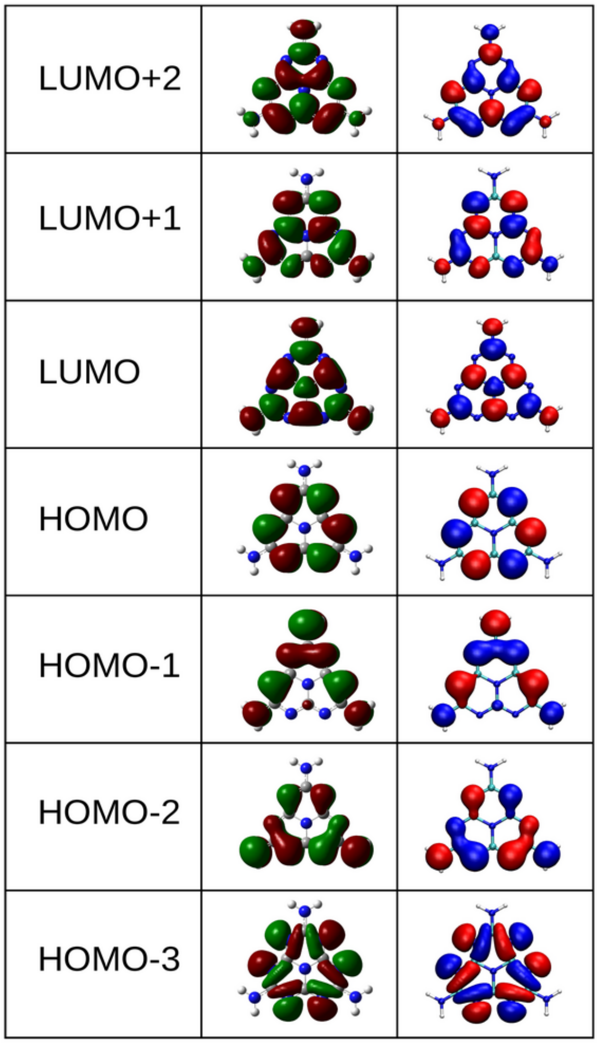

Figure S8: Isosurfaces of the frontier molecular orbital wavefunctions of the melem (MLM) molecule: Kohn-Sham MOs obtained using the DFT-wB97xD/6-31g(d,p) method (green-red) and the parametrized e-Hückel model Hamiltonian (blue-red).

Table S8: Optimized extended Hückel parameters for the melem (MLM) molecule.

**Symbol**	**EHsymbol**	**Residue**	**NoAt**	**Nvalen**	**n**	**spdf**	**IP**	**zeta1**	**k_WH**
N	NA	MLM	7	5	2	s	-26.01979	2.079830	1.729480
N	NA	MLM	7	5	2	p	-15.26818	2.041603	2.257440
C	CP	MLM	6	4	2	s	-20.38691	1.949468	1.305237
C	CP	MLM	6	4	2	p	-10.50847	2.358813	3.252546
N	NB	MLM	7	5	2	s	-26.46465	1.883892	1.286840
N	NB	MLM	7	5	2	p	-12.15770	2.105099	4.045040
C	CA	MLM	6	4	2	s	-20.34621	2.135495	1.642137
C	CA	MLM	6	4	2	p	-10.49715	2.213987	4.545426
N	NH	MLM	7	5	2	s	-26.27534	1.141419	2.837650
N	NH	MLM	7	5	2	p	-14.25801	2.575447	3.442110
H	HN	MLM	1	1	1	s	-13.74962	1.395763	1.917330

Table S9: Electronic transitions with corresponding excitation energies and oscillator strengths (f) for the MLM molecule in the optimized ground-state geometry.

**Turbomole / ADC(2) cc-pVDZ**				**e-Hückel model**
**Transition**	**E (eV)**	**f**	**MO Transition**	**E (eV)**
$S_0 \rightarrow S_1$	3.82	0	H $\rightarrow$ L	3.82
$S_0 \rightarrow S_2$	5.10	0.3424	H $\rightarrow$ L+1	5.09
$S_0 \rightarrow S_3$	5.10	0.3424	H $\rightarrow$ L+2	5.10
$S_0 \rightarrow S_4$	5.58	0.541	H-1 $\rightarrow$ L	5.59
$S_0 \rightarrow S_5$	5.58	0.541	H-2 $\rightarrow$ L	5.59
$S_0 \rightarrow S_6$	6.03	0	H-3 $\rightarrow$ L	6.02

4.3 Exited-State Dynamics of Single Melem Molecule

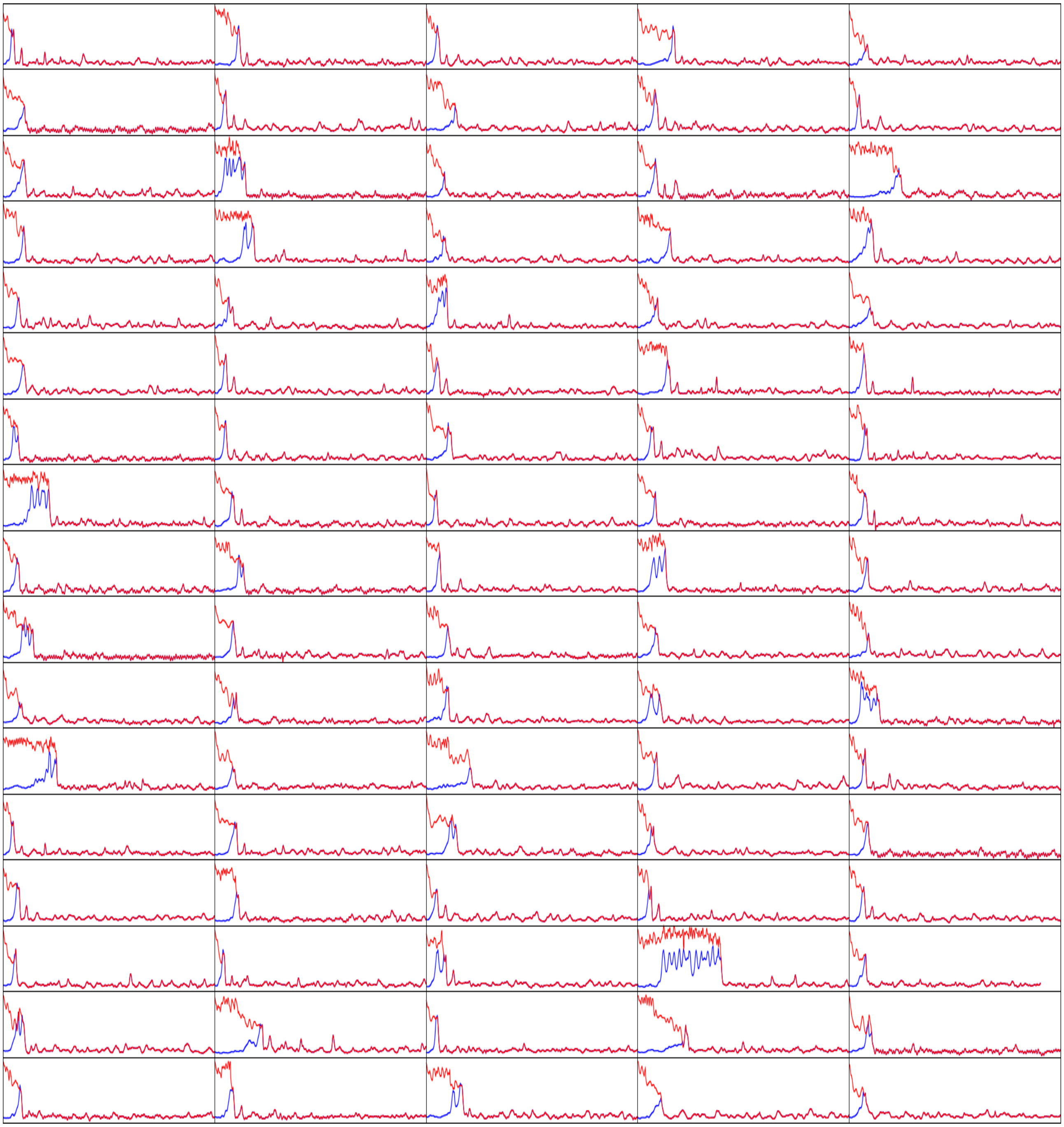

Figure S9: Excited-state nonadiabatic molecular dynamics trajectories were generated following the $S_0 \rightarrow S_2$ excitation of the of the melem (MLM) molecule. For all the graphs the vertical axis describes energy in the range $\varepsilon = [-10, -3.8]$ eV whereas the horizontal axis describes the time in the range $t = [0, 1]$ ps. The curves represent the expectation values of the energy, $\varepsilon_\alpha = \langle \psi_\alpha(t)|\hat{H}|\psi_\alpha(t)\rangle$, for the electron ($\alpha =$ el, red) and the hole ($\alpha =$ hl, blue).

4.4 Exited-State Dynamics of 6-Melem Aggregate

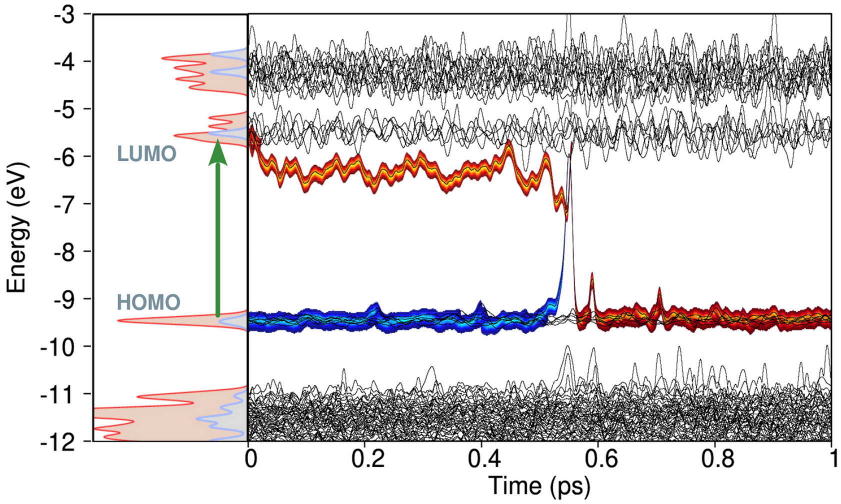

Figure S10: Dynamics of the photoexcited electron-hole pair in the 6-MLM aggregate. The graph illustrates the evolution of molecular orbital (MO) energies (black lines) over time, computed dynamically during the excited-state nonadiabatic molecular dynamics (XS-NAMD) simulation. The thick orange trace represents the electronic MO occupation, while the blue trace depicts the hole wavefunction occupation. At $t \approx 0.6$ ps, the blue and orange traces converge, marking the occurrence of electron-hole nonradiative recombination. This XS-NAMD simulation corresponds to the one highlighted with an asterisk in Figure S11.

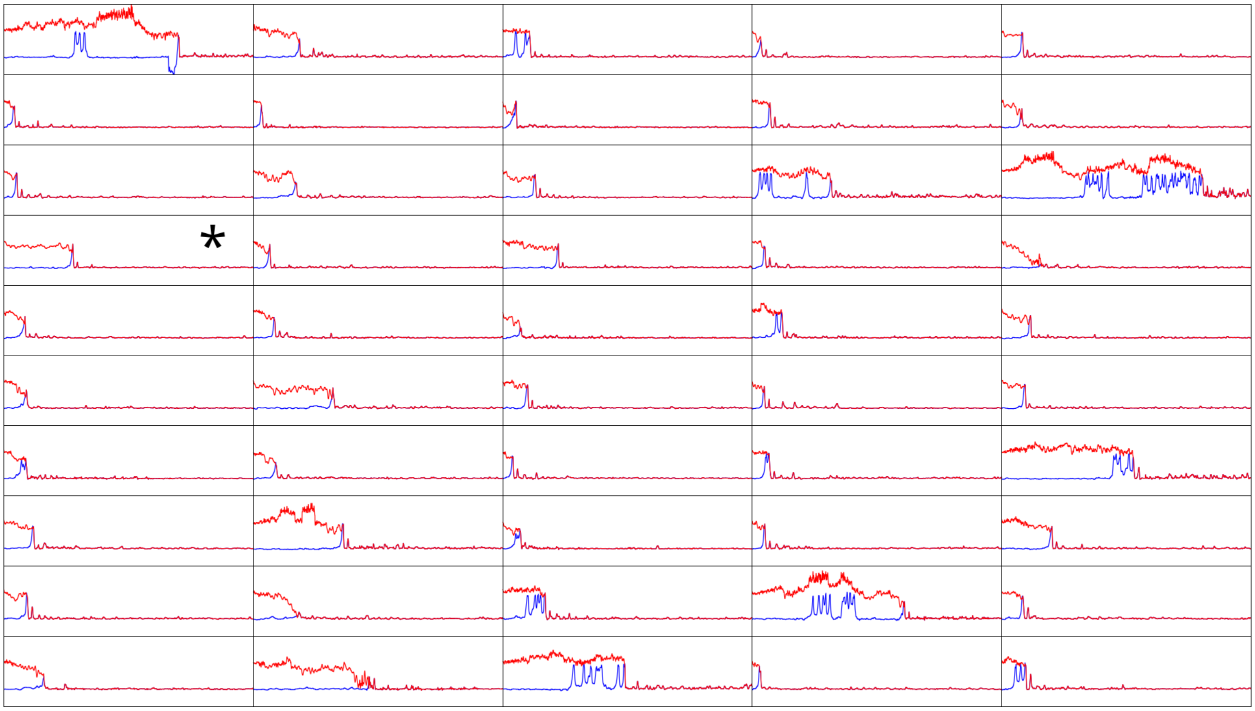

Figure S11: Fifty excited-state nonadiabatic molecular dynamics trajectories were generated following the $S_0 \rightarrow S_1$ excitation of the central melem molecule of the 6-MLM aggregate. For all the graphs the vertical axis describes energy in the range $\varepsilon = [-12, -1.7]$ eV whereas the horizontal axis describes the time in the range $t = [0, 2]$ ps. The curves represent the expectation values of the energy, $\varepsilon_\alpha = \langle\psi_\alpha(t)|\hat{H}|\psi_\alpha(t)\rangle$, for the electron ($\alpha$ = el, red) and the hole (α = hl, blue). The graph with an asterisk is presented in detail in Figure S10.

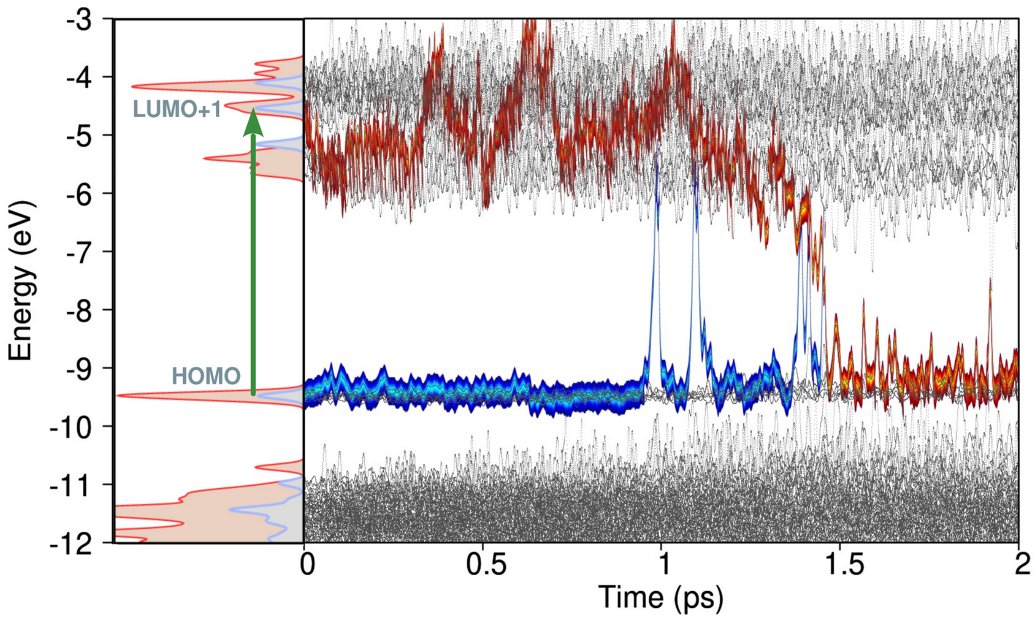

Figure S12: Dynamics of the photoexcited electron-hole pair in the 6-MLM aggregate following the $S_0 \rightarrow S_2$ excitation of the central melem molecule of the 6-MLM aggregate. The graph illustrates the evolution of molecular orbital (MO) energies (black lines) over time, computed dynamically during the excited-state nonadiabatic molecular dynamics (XS-NAMD) simulation. The thick orange trace represents the electronic MO occupation, while the blue trace depicts the hole wavefunction occupation. At $t \approx 1.1$ ps, the blue and orange traces converge, marking the occurrence of electron-hole nonradiative recombination. This XS-NAMD simulation corresponds to the one highlighted with an asterisk in Figure S15.

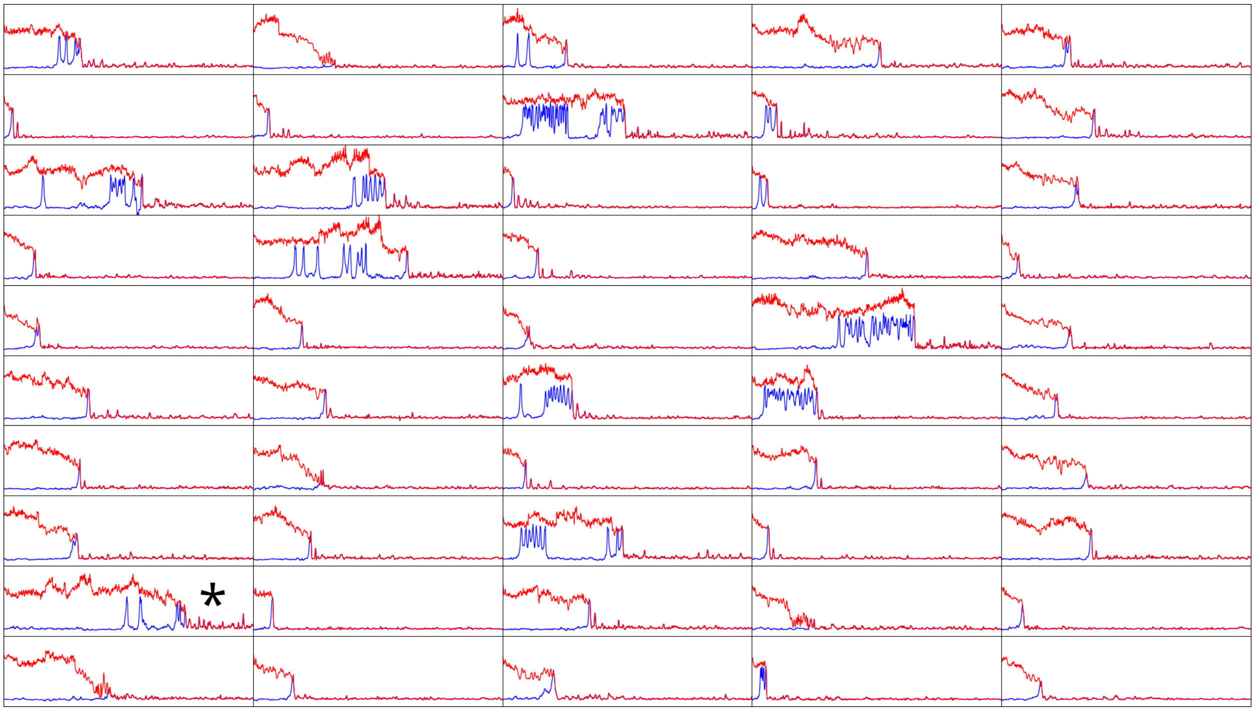

Figure S13: Fifty excited-state nonadiabatic molecular dynamics trajectories were generated following the $S_0 \rightarrow S_2$ excitation of the central melem molecule of the 6-MLM aggregate. For all the graphs the vertical axis describes energy in the range $\varepsilon = [-10.3, -2.1]$ eV whereas the horizontal axis describes the time in the range $t = [0, 2]$ ps. The curves represent the expectation values of the energy, $\varepsilon_\alpha = \langle \psi_\alpha(t)|\hat{H}|\psi_\alpha(t)\rangle$, for the electron ($\alpha$ = el, red) and the hole (α = hl, blue).

5 Contrasting Heptazine and Melem Relaxations

5.1 Effect of Aggregation on Molecular Mobility

In Figure S14, we present an analysis of fifty excited-state MD trajectories, comparing the bending and twisting degrees of freedom of the melem molecule in both the isolated (MLM-gas) and 6-MLM aggregate (MLM-pack) environments.

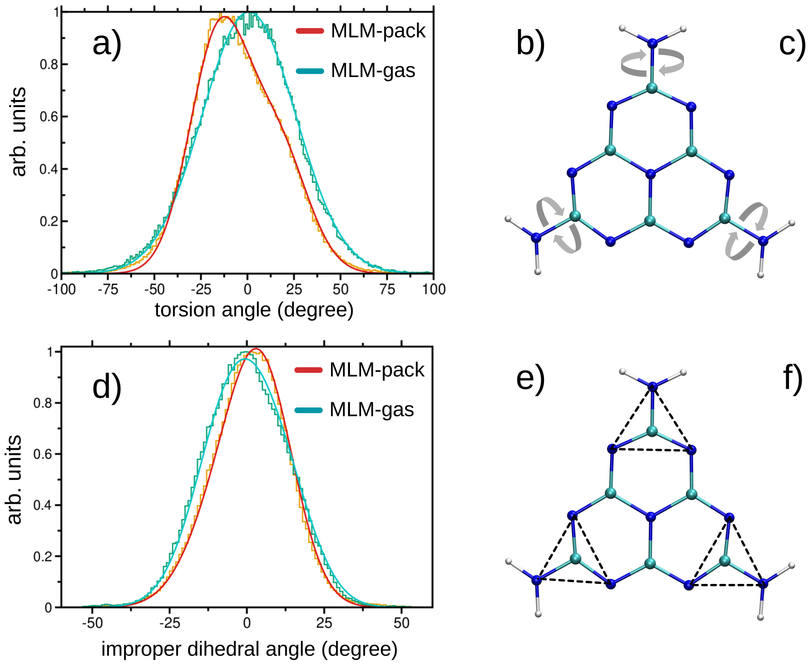

c)

$$y = A_1 e^{-\frac{(x-\mu_1)^2}{2\sigma_1^2}} + A_2 e^{-\frac{(x-\mu_2)^2}{2\sigma_2^2}}$$

parms	1-MLM	6-MLM
A_1	1.0	0.89
μ_1	0.8	-15.50
σ_1	25.14	15.78
A_2	–	0.48
μ_2	–	16.00
σ_2	–	16.29

f)

parms	1-MLM	6-MLM
A_1	0.85	0.85
μ_1	-3.78	5.13
σ_1	11.91	10.28
A_2	0.33	0.31
μ_2	12.28	-8.69
σ_2	10.31	11.00

Figure S14: Effect of aggregation on molecular mobility. (a) Histogram of the torsion angles of the NH_2 groups in the melem molecule in the gas phase (MLM-gas) and in the central MLM molecule within the 6-MLM aggregate (MLM-pack). (b) Illustration of the torsional degree of freedom. (c) Parameters used to fit the torsion angle histogram. (d) Histogram of the improper dihedral angles in the melem molecule in the gas phase and in the central MLM molecule within the 6-MLM aggregate. (e) Illustration of the improper dihedral degree of freedom. (f) Parameters used to fit the dihedral angle histogram.

In the upper panel (Figures S14a–S14c), we examine the twisting motion of the three NH_2 groups in the melem molecule. Figure S14b illustrates the geometric definition of the torsional angle, while Figure S14a shows the histogram of torsion angles obtained from the trajectories. The corresponding Gaussian fits are shown in Figure S14c. The results indicate that the isolated melem molecule exhibits greater rotational freedom of the NH_2 groups, with

a broad distribution of torsion angles well described by a single Gaussian with a standard deviation of approximately $\sigma \approx 25°$. In contrast, the twisting motion of the NH_2 groups in the central MLM molecule within the aggregate is significantly restricted, as evidenced by a narrower distribution fitted by two Gaussian components in a 2:1 ratio, each with a standard deviation of $\sigma \approx 16°$. This suggests that molecular packing in the aggregate imposes constraints on NH_2 torsional flexibility. In the lower panel (Figures S14d–S14f), we analyze the pyramidalization degree of freedom, as illustrated in Figure S14e. In this case, the comparison shows no significant difference between the isolated and aggregated environments.

5.2 Internal Conversion Pathways in Heptazine and Melem

In the upper panel, Figure S15 presents the free relaxation dynamics of heptazine (HTZ) (Figure S15a) and melem (MLM) (Figure S15b) from the S_1 to the S_0 state, starting from the ground-state optimized geometry with atoms initially at rest. The thick orange trace represents the electronic MO occupation, while the blue trace indicates the hole wavefunction occupation. The black lines show the evolution of MO energies over time, computed dynamically during the excited-state non-adiabatic molecular dynamics (NAMD) simulation. ADC(2) calculations reveal that the S_1 state is predominantly a HOMO–LUMO excitation (approximately 96%), which supports the validity of our semiempirical single-particle description of the relaxation dynamics.

The relaxation dynamics show that melem undergoes faster $S_1 \rightarrow S_0$ internal conversion than heptazine, consistent with the trends discussed in the main text. To rationalize this behavior, Figure S15c displays the potential energy profiles of HTZ (solid lines) and MLM (dashed lines) along a linear interpolation from the planar optimized geometry to their respective potential energy crossing points. This analysis reveals that the S_1 potential energy profile of MLM is significantly steeper than that of HTZ. Consequently, once a slight energy barrier is overcome, the MLM molecule experiences a stronger driving force toward the

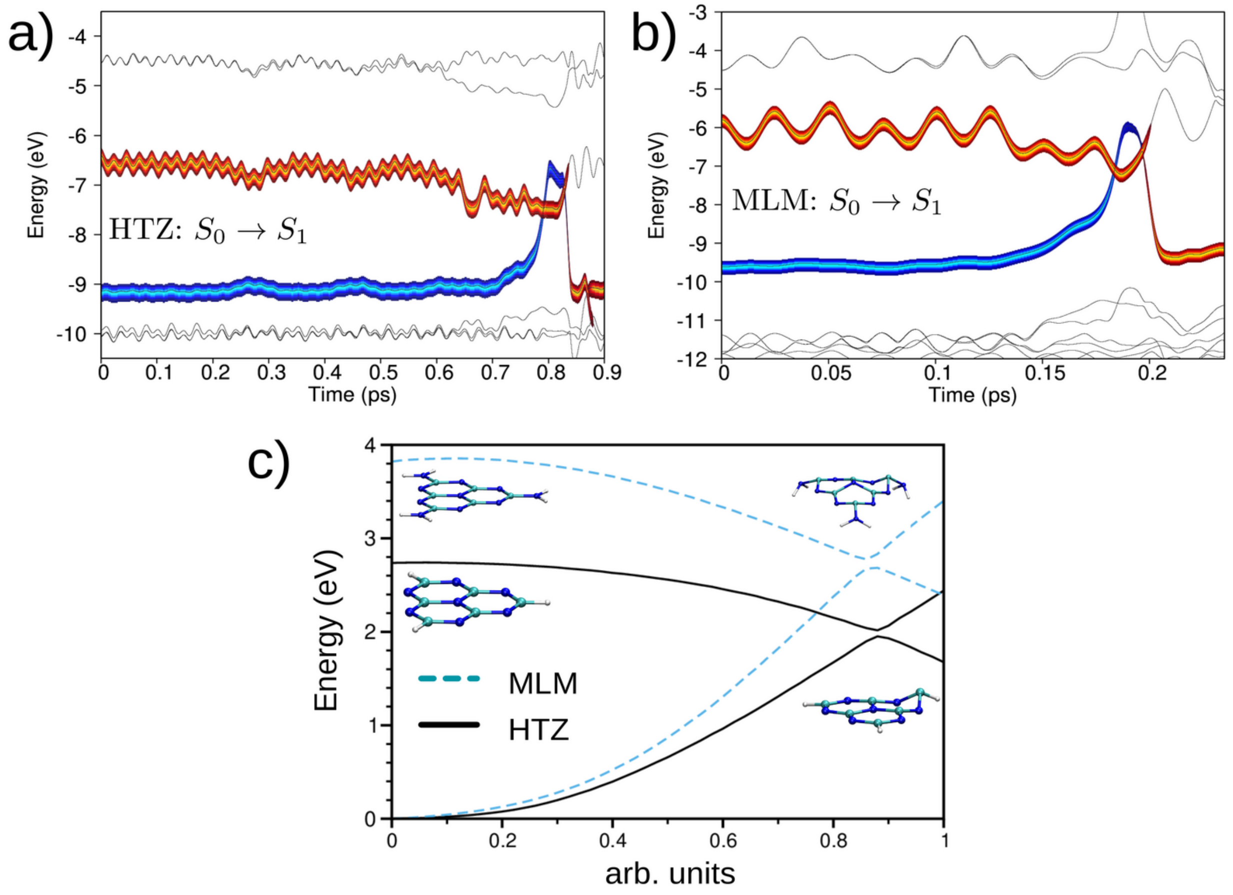

Figure S15: (a) Free relaxation dynamics of heptazine (HTZ) from the S_1 to the S_0 state, starting from the ground-state optimized geometry with atoms initially at rest. (b) Same as (a), but for the melem (MLM) molecule. (c) Potential energy profiles of HTZ (solid lines) and MLM (dashed lines) along a linear interpolation between the planar optimized geometry to the respective potential energy crossing points. Insets depict representative molecular geometries along the relaxation paths.

crossing region, resulting in a faster internal conversion.

Furthermore, Figure S15 highlights the distinction between free relaxation dynamics—which captures the real-time evolution of the system on a multidimensional excited-state potential energy surface—and static potential energy scans along predefined generalized coordinates.

References

(1) Zhu, C.; Nangia, S.; Jasper, A. W.; Truhlar, D. G. Coherent switching with decay of mixing: An improved treatment of electronic coherence for non-Born–Oppenheimer trajectories. *J. Chem. Phys.* **2004**, *121*, 7658–7670.

(2) Wang, J.; Wolf, R. M.; Caldwell, J. W.; Kollman, P. A.; Case, D. A. Development and testing of a general amber force field. *J. Comput. Chem.* **2004**, *25*, 1157–1174.

(3) Ehrmaier, J.; Rabe, E. J.; Pristash, S. R.; Corp, K. L.; Schlenker, C. W.; Sobolewski, A. L.; Domcke, W. Singlet–Triplet Inversion in Heptazine and in Polymeric Carbon Nitrides. *J. Phys. Chem. A* **2019**, *123*, 8099–8108.

(4) de Silva, P. Inverted Singlet–Triplet Gaps and Their Relevance to Thermally Activated Delayed Fluorescence. *J. Phys. Chem. Lett.* **2019**, *10*, 5674–5679.

(5) Sandoval-Salinas, M. E.; Ricci, G.; Pérez-Jiménez, A. J.; Casanova, D.; Olivier, Y.; Sancho-García, J. C. Correlation vs. exchange competition drives the singlet–triplet excited-state inversion in non-alternant hydrocarbons. *Phys. Chem. Chem. Phys.* **2023**, *25*, 26417–26428.

(6) Ricci, G.; San-Fabián, E.; Olivier, Y.; Sancho-García, J. C. Singlet-Triplet Excited-State Inversion in Heptazine and Related Molecules: Assessment of TD-DFT and ab initio Methods. *ChemPhysChem* **2021**, *22*, 553–560.

(7) Pollice, R.; Friederich, P.; Lavigne, C.; dos Passos Gomes, G.; Aspuru-Guzik, A. Organic molecules with inverted gaps between first excited singlet and triplet states and appreciable fluorescence rates. *Matter* **2021**, *4*, 1654–1682.
